# Biogeographical patterns and speciation of the genus *Pinguicula* (Lentibulariaceae) inferred by phylogenetic analyses

**DOI:** 10.1371/journal.pone.0252581

**Published:** 2021-06-07

**Authors:** Hiro Shimai, Hiroaki Setoguchi, David L. Roberts, Miao Sun

**Affiliations:** 1 Durrell Institute of Conservation and Ecology, School of Anthropology and Conservation, University of Kent, Canterbury, Kent, United Kingdom; 2 Graduate School of Human and Environmental Studies, Kyoto University, Sakyo-ku, Kyoto, Japan; 3 Florida Museum of Natural History, University of Florida, Gainesville, Florida, United States of America; Laboratoire de Biologie du Développement de Villefranche-sur-Mer, FRANCE

## Abstract

Earlier phylogenetic studies in the genus *Pinguicua* (Lentibulariaceae) suggested that the species within a geographical region was rather monophyletic, although the sampling was limited or was restricted to specific regions. Those results conflicted with the floral morphology-based classification, which has been widely accepted to date. In the current study, one nuclear ribosomal DNA (internal transcribed spacer; ITS) and two regions of chloroplast DNA (*matK* and *rpl32-trnL*), from up to ca. 80% of the taxa in the genus *Pinguicula*, covering all three subgenera, were sequenced to demonstrate the inconsistency and explore a possible evolutionary history of the genus. Some incongruence was observed between nuclear and chloroplast topologies and the results from each of the three DNA analyses conflicted with the morphology-based subgeneric divisions. Both the ITS tree and network, however, corresponded with the biogeographical patterns of the genus supported by life-forms (winter rosette or hibernaculum formation) and basic chromosome numbers (haploidy). The dormant strategy evolved in a specific geographical region is a phylogenetic constraint and a synapomorphic characteristic within a lineage. Therefore, the results denied the idea that the Mexican group, morphologically divided into the three subgenera, independently acquired winter rosette formations. Topological incongruence among the trees or reticulations, indicated by parallel edges in phylogenetic networks, implied that some taxa originated by introgressive hybridisation. Although there are exceptions, species within the same geographical region arose from a common ancestor. Therefore, the classification by the floral characteristics is rather unreliable. The results obtained from this study suggest that evolution within the genus *Pinguicula* has involved; 1) ancient expansions to geographical regions with gene flow and subsequent vicariance with genetic drift, 2) acquirement of a common dormant strategy within a specific lineage to adapt a local climate (i.e., synapomorphic characteristic), 3) recent speciation in a short time span linked to introgressive hybridisation or multiplying the ploidy level (i.e., divergence), and 4) parallel evolution in floral traits among lineages found in different geographical regions (i.e., convergence). As such, the floral morphology masks and obscures the phylogenetic relationships among species in the genus.

## Introduction

The family Lentibulariaceae, consisting of three carnivorous genera, *Genlisea* A.St.-Hill. (ca. 30 species), *Pinguicula* L. (ca. 100 spp.), and *Utricularia* L. (> 200 spp.), are widespread herbs in wetlands, from tropical to cold regions [1]. Species from the genus *Pinguicula* (butterwort) essentially form a basal rosette with adhesive leaves, a short stem, a true root system, and simple ebracteate scapes which bear a terminal flower at each apex [2–5], and thus the genus is a well-defined taxonomic group both morphologically [6] and phylogenetically [7, 8]; being a sister group of the other two genera.

**Fig 1:**
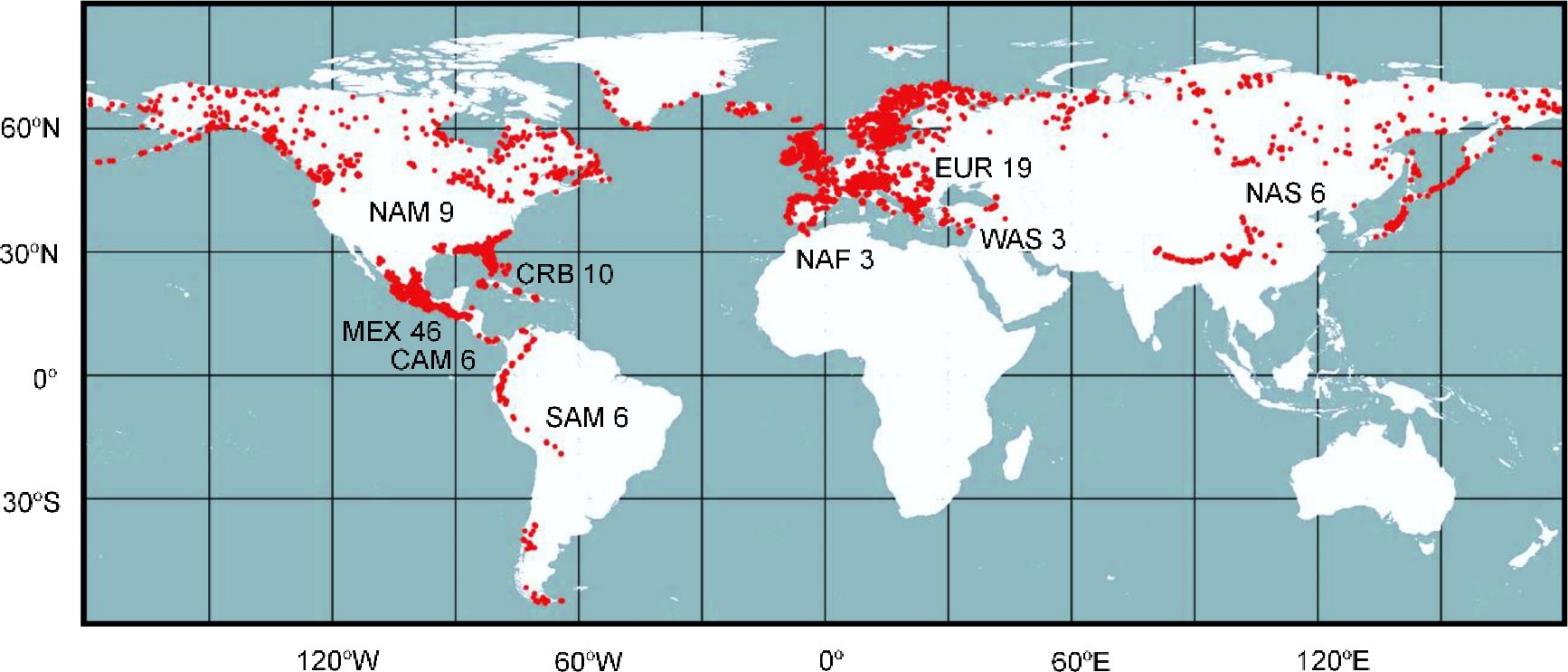
Distribution of *Pinguicula*. Red dots indicate the distribution of *Pinguicula* based on over 7,000 herbarium specimen examinations by Shimai [9]. The distribution area is divided into nine regions: CAM = Central America; CRB = the Caribbean; EUR = Europe; MEX = Mexico; NAF = North Africa (Morocco); NAM = North America; NAS = Northeastern Asia; SAM = South America; WAS = Western Asia (for more details, see the Materials and Methods section). The number after region code indicates the number of species in each region (some species are distributed in two or more regions). The map was made with Natural Earth (https://www.naturalearthdata.com/).

The distribution of the genus *Pinguicula* encompasses Eurasia, North to South America, the Caribbean, and Morocco (Fig 1) [1, 3, 5, 9]. Although the genus presents an extensive geospatial distribution range, they are commonly restricted to nutrient-poor wet soils, such as bogs (acidic soils often with peat or sphagnums), fens (alkaline soils often with calcareous or serpentinous rocks), stream sides, pond margins, rock faces with dripping, splashing water, or water films [5, 6, 10, 11], as well as semidried soils with fogs and high precipitations providing moisture over the soil and plant body [12]. Species in the genus are terrestrial, lithophytic, or rarely epiphytic. Their microhabitat is usually confined to north-facing slopes, gorges, or forests with limited light intensity to avoid heat [13–18]. Average monthly temperature is also one of the factors restricting the distribution [19]. Population size at each microhabitat is often small or sparse.

Casper [3] recognised 46 species and divided them into three subgenera, *Isoloba* Barnhart, *Pinguicula*, and *Temnoceras* Barnhart, based mainly on their flower colour and corolla shape, composed of a two-lobed upper lip and a three-lobed lower lip. Hence, the subgenus *Isoloba* possesses subactinomorphic corollas formed by substantially equal shapes of five lobes often emarginate to bifid at the tip, the subgenus *Pinguicula* possesses zygomorphic corollas formed by two small upper lobes and three large lower lobes (often the mid-lobe is larger than laterals) usually darker in colour (e.g., purple or violet) while the subgenus *Temnoceras* are paler in colour (e.g., faint purple) or white. Casper [3] divided the three subgenera into a further 12 sections incorporating many subsections and series since the subgeneric delimitation did not consistently embrace the life-forms or chromosome numbers.

**Fig 2:**
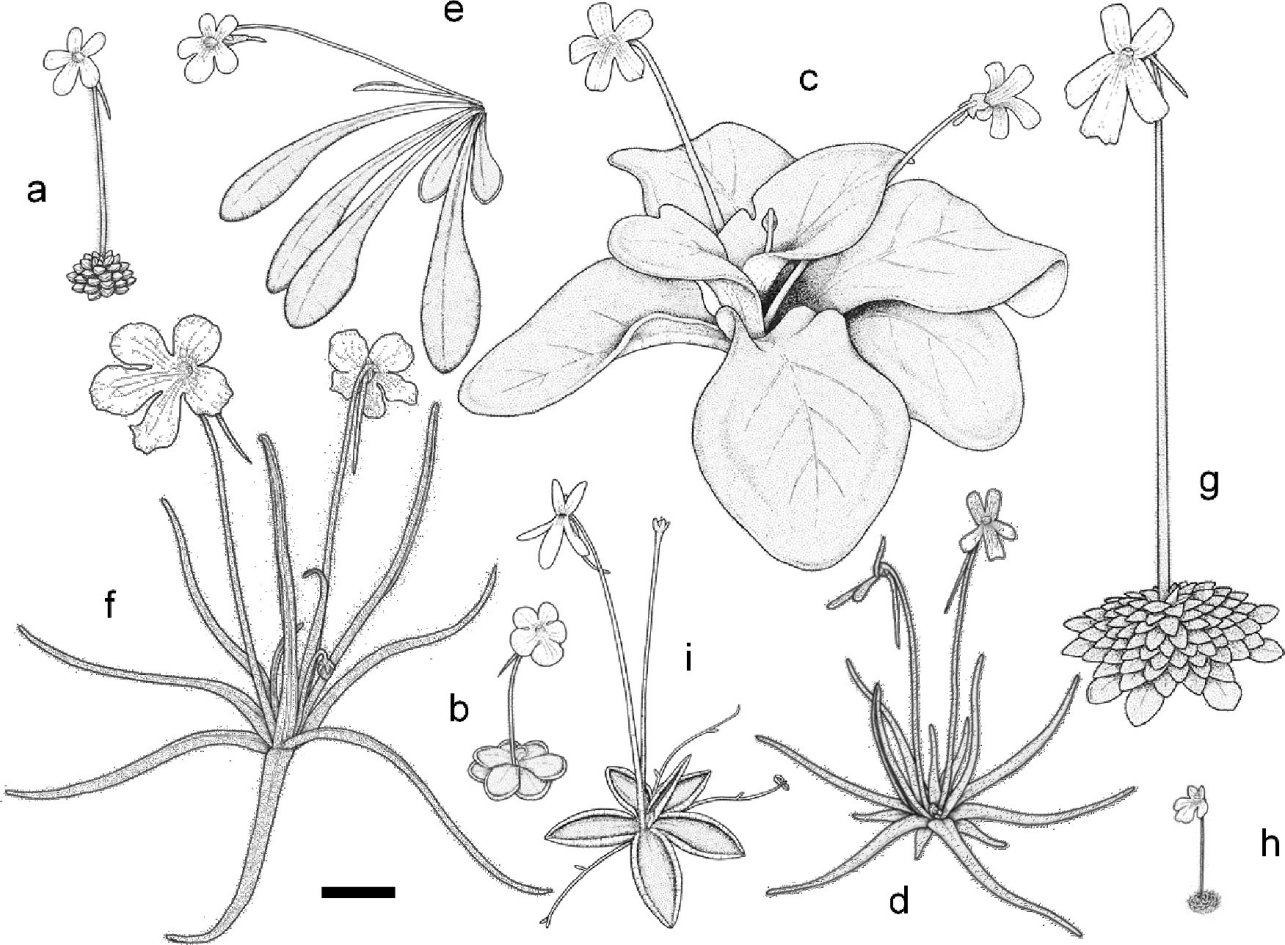
Morphological diversity in Mexican *Pinguicula*. Some representative Mexican species are illustrated: (a) *P*. *crassifolia* (winter rosette); (b) *P*. *cyclosecta*; (c) *P*. *gigantea*; (d) *P*. *gypsicola*; (e) *P*. *laxifolia*; (f) *P*. *moctezumae*; (g) *P*. *moranensis* (winter rosette); (h) *P*. *nivalis* (winter rosette); (i) *P*. *orchidioides*. Subgenera *sensu* Casper: *Isoloba* (c); *Pinguicula* (a, b, d, e, f, g, i); *Temnoceras* (h). Bar indicates ca. 30 mm. Drawn by H. Shimai.

Since then, a number of additional species have been described mainly from Europe (e.g., [20–23]), Mexico (e.g., [13, 17, 24–26]), and Cuba (e.g., [27]). The International Plant Names Index [28] lists over 200 specific and infraspecific taxon names of *Pinguicula*. Some of them are considered to be synonymous with other taxa [29–31] (S1 Appendix); therefore, taxonomists normally recognise from 90 to over 100 species in the genus [9, 32–35]. As a result, the number of species has doubled since Casper’s [3] taxonomic treatment. In this current study, the three subgenera *sensu* Casper are discussed rather than his fractionated infrasubgeneric ranks. Although the structure of the plant is fundamentally uniform [6], considerable morphological diversity among species is seen, not only in the flower but also in the leaf shape and rosette size, particularly in Mexico (Fig 2), which harbours over 40 species [33, 34], ca. 90% of which are endemic [36]. Based upon the floral characteristics, Mexican species are divided into the three subgenera derived from multiple ancestors [3].

The number of chromosomes has been reported from a series of *Pinguicula* taxa (e.g., [3, 37, 38]); however, the number itself has little correspondence to the classification *sensu* Casper [3]. Beyond the morphological classification, life-forms and distribution areas are often used to group species [39–41]. Those groups are 1) species in Mexico which form winter rosettes (often lenticular to subglobose in shape) with numerous small succulent leaves densely surrounding the growing point to resist dry winter; 2) taxa in mild to cold or boreal (hereafter temperate) regions of the Northern Hemisphere which form hibernacula (often ovoid) with scale-like cymbiform leaves tightly overlapping in layers around the growing point to endure low temperature in winter; and 3) taxa in warmer or low-altitude subtropical regions, e.g., in the southeastern USA, the Caribbean, and South America, which grow throughout the year (i.e., which are homophyllous). A few other homophyllous species are also distributed in Western Eurasia, Morocco, Mexico, and Central America. Although the temperate climate extends to Mexico, Mexican species present apparent distribution gaps with species in the temperate Northern Hemisphere or the southeastern USA. Thus, the subgeneric division does not necessarily correspond with those traits and geographical distributions.

Apart from the morphology-based classification, previous phylogenetic studies of the genus *Pinguicula* including different numbers of species and DNA regions attempted to infer the relationships of the species [32, 35, 42, 43]. An analysis with *trnK* and *matK* (hereafter *matK*) in 42 taxa, performed by Cieslak *et al*. [32] and updated by Beck *et al*. [35], showed that each of the three subgenera was polyphyletic and lineages were geographically dependent. Degtjareva *et al*. [42] and Kondo & Shimai [43] analysed taxa mainly from the temperate Northern Hemisphere using the internal transcribed spacer (ITS) region, and they showed that taxa forming rootless hibernacula in the section *Pinguicula* were monophyletic. Shimai & Kondo [44] analysed the ITS (ITS-1, 5.8S, and ITS-2) regions of 36 species from Mexico and Central America and suggested that the species were monophyletic, although Casper [3] had divided them into three subgenera. Overall, those phylogenetic analyses were not consistent with the morphology-based classification. It is hypothesised in the current study that a lineage in each geographical region is rather monophyletic, but the floral characteristic masks the phylogenetic relationships among the species and the evolutionary pathway of the genus.

The infrageneric treatment was recently rearranged by Fleischmann & Roccia [36] based on *matK* as follows. The subgenus *Isoloba* includes the sections *Isoloba* Casper, *Cardiophyllum* Casper, *Pumiliformis* (Casper) Roccia & A.Fleischm., and *Ampullipalatum* Casper; the subgenus *Pinguicula* contains the section *Pinguicula* alone; and the subgenus *Temnoceras* contains the sections *Temnoceras* Casper, *Micranthus* Casper, *Nana* Casper, and *Heterophylliformis* (Casper) A.Fleischm. & Roccia. Nevertheless, Fleischmann and Roccia [36] admitted that the subgenus *Temnoceras sensu* Fleischmann & Roccia was not clearly resolved by *matK*.

Regardless of the taxonomy, the taxa are often grouped in accordance with life-forms associated with geographical regions and climates where they can be found. A question emerges as to whether, or not, such life-forms resulted from convergence that took place in different lineages within the same geographical region, as the floral morphology-based classification would suggest. In addition, if hybridisation was involved in the speciation, the relationship among species would not be tree-like but would be reticulations visualised by phylogenetic networks. In our present study, ITS in nuclear ribosomal DNA (nrDNA), and *matK* and *rpl32-trnL* in chloroplast DNA (cpDNA) are analysed to reconstruct phylogenetic trees and networks, and to explore further the evolutionary pathway of the genus *Pinguicula*.

## Materials and methods

### DNA extraction and amplification

Sampled taxa and their voucher information are summarised in Table 1. The number of sampled taxa analysed for ITS, *matK*, and *rpl32-trnL* were 79, 69, and 69, respectively. The *matK* analysis included 39 sequences from Cieslak *et al*. [32] and Beck *et al*. [35] deposited in the International Nucleotide Sequence Database (INSD; http://www.insdc.org/); therefore, the total number of *Pinguicula* taxa listed in Table 1 is 82. Some taxa sampled for the present study may be synonymous with other species; however, the original scientific names were used to be consistent with the registered names in the INSD. For DNA extraction, either fresh or dried leaves were used, depending on the availability of samples. Fresh leaves were obtained from live plants while dried leaves were collected from herbarium specimens.

**Table 1:** Sampled taxa, accession numbers, and voucher specimens.

Taxon	Accession number			Specimen number and herbarium code
	ITS	*matK*	*rpl32-trnL*	
***Pinguicula acuminata***	AB199751	DQ010652	LC348618	Kondo & Shimai 5757-LPCGS (HIRO)
***P*. *agnata***	AB199752	AF531782	n/a	Kondo & Shimai 5758-LPCGS (HIRO)
***P*. *albida***	AB212095	LC348432	LC348619	Kondo & Shimai 5788-LPCGS (HIRO)
***P*. *alpina***	AB198341	AF531783	LC348620	Kondo & Shimai 5734-LPCGS (HIRO)
***P*. *antarctica***	AB212096	DQ010653	LC348621	Kondo & Shimai 716-LPCGS (HIRO)
***P*. *balcanica* subsp. *balcanica***	AB198342	n/a	n/a	Kondo & Shimai 5738-LPCGS (HIRO)
***P*. *balcanica* subsp. *pontica***	LC348695	n/a	LC348622	Shimai s.n. VS 1252249 (TNS)
***P*. *benedicta***	AB212097	LC348433	LC348623	Kondo & Shimai 715-LPCGS (HIRO)
***P*. *bissei***	AB212098	LC348434	LC348624	Kondo & Shimai 5790-LPCGS (HIRO)
***P*. *bohemica***	AB198343	LC348435	LC348625	Kondo & Shimai 5739-LPCGS (HIRO)
***P*. *caerulea***	AB212099	n/a	LC348626	Kondo & Shimai 5791-LPCGS (HIRO)
***P*. *calyptrata***	AB212100	FM200225	LC348627	Kondo & Shimai 717-LPCGS (HIRO)
***P*. *chilensis***	AB212101	n/a	LC348628	Kondo & Shimai 718-LPCGS (HIRO)
***P*. *chuquisacensis***	n/a	FM200223	n/a	
***P*. *colimensis***	AB199753	LC348436	LC348629	Kondo & Shimai 5759-LPCGS (HIRO)
***P*. *conzattii***	AB199754	LC348437	LC348630	Kondo & Shimai 709-LPCGS (HIRO)
***P*. *corsica***	AB198344	AF531784	LC348631	Kondo & Shimai 5740-LPCGS (HIRO)
***P*. *crassifolia***	AB199755	LC348438	LC348632	Kondo & Shimai 5760-LPCGS (HIRO)
***P*. *crenatiloba***	LC348696	n/a	LC348633	Shimai s.n. VS 1266600 (TNS)
***P*. *crystallina***	AB198363	n/a	LC348634	Kondo & Shimai 5753-LPCGS (HIRO)
***P*. *cubensis***	AB212102	LC348439	LC348635	Kondo & Shimai 5794-LPCGS (HIRO)
***P*. *cyclosecta***	AB199756	LC348440	LC348636	Kondo & Shimai 5761-LPCGS (HIRO)
***P*. *debbertiana***	AB199757	LC348441	n/a	Kondo & Shimai 5762-LPCGS (HIRO)
***P*. *dertosensis***	AB198345	LC348442	LC348637	Kondo & Shimai 5741-LPCGS (HIRO)
***P*. *ehlersiae***	AB199758	LC348443	LC348638	Kondo & Shimai 5763-LPCGS (HIRO)
***P*. *elongata***	AB212103	FM200224	LC348639	Kondo & Shimai 718-LPCGS (HIRO)
***P*. *emarginata***	AB199759	AF531785	LC348640	Kondo & Shimai 5764-LPCGS (HIRO)
***P*. *esseriana***	AB199760	DQ010656	LC348641	Kondo & Shimai 5765-LPCGS (HIRO)
***P*. *filifolia***	AB212104	AF531786	LC348642	Kondo & Shimai 5795-LPCGS (HIRO)
***P*. *fiorii***	AB198346	AF531787	LC348643	Kondo & Shimai 5742-LPCGS (HIRO)
***P*. *gigantea***	AB199761	AF531789	LC348644	Kondo & Shimai 5766-LPCGS (HIRO)
***P*. *gracilis***	AB199762	AF531790	LC348645	Kondo & Shimai 5767-LPCGS (HIRO)
***P*. *grandiflora***	AB198347	AF531791	LC348646	Kondo & Shimai 701-LPCGS (HIRO)
***P*. *gypsicola***	AB199763	LC348444	n/a	Kondo & Shimai 5768-LPCGS (HIRO)
***P*. *hemiepiphytica***	AB199764	LC348445	LC348647	Kondo & Shimai 5769-LPCGS (HIRO)
***P*. *heterophylla***	AB199765	n/a	LC348648	Kondo & Shimai 5770-LPCGS (HIRO)
***P*. *hirtiflora***	AB198364	DQ010654	n/a	Kondo & Shimai 5754-LPCGS (HIRO)
***P*. *ibarrae***	AB251603	LC348446	LC348649	Kondo & Shimai 5771-LPCGS (HIRO)
***P*. *immaculata***	AB199766	LC348447	LC348650	Kondo & Shimai 5772-LPCGS (HIRO)
***P*. *involuta***	n/a	FM200226	n/a	
***P*. *ionantha***	AB212105	LC348448	LC348651	Kondo & Shimai 5796-LPCGS (HIRO)
***P*. *jackii* var. *jackii***	AB212106	n/a	n/a	Kondo & Shimai 5797-LPCGS (HIRO)
***P*. *jackii* var. *parviflora***	AB212107	LC348449	LC348652	Kondo & Shimai 5798-LPCGS (HIRO)
***P*. *jaumavensis***	AB199767	LC348450	LC348653	Kondo & Shimai 5773-LPCGS (HIRO)
***P*. *laueana***	AB199768	DQ010659	LC348654	Kondo & Shimai 5774-LPCGS (HIRO)
***P*. *leptoceras***	AB198349	AF531792	LC348655	Kondo & Shimai 5744-LPCGS (HIRO)
***P*. *lignicola***	AB300151	n/a	n/a	Kondo & Shimai 5803-LPCGS (HIRO)
***P*. *lilacina***	AB199769	LC348452	LC348656	Kondo & Shimai 5775-LPCGS (HIRO)
***P*. *longifolia* subsp. *caussensis***	AB198350	AF531794	LC348657	Kondo & Shimai 5745-LPCGS (HIRO)
***P*. *longifolia* subsp. *longifolia***	AB198351	AF531793	LC348658	Kondo & Shimai 702-LPCGS (HIRO)
***P*. *longifolia* subsp. *reichenbachiana***	AB198352	DQ010660	LC348659	Kondo & Shimai 5746-LPCGS (HIRO)
***P*. *lusitanica***	AB198365	DQ010661	LC348660	Kondo & Shimai 5752-LPCGS (HIRO)
***P*. *lutea***	AB212108	DQ010662	LC348661	Kondo & Shimai 5799-LPCGS (HIRO)
***P*. *macroceras***	AB198353	AF531796	LC348662	Kondo & Shimai 5747-LPCGS (HIRO)
***P*. *macrophylla***	AB199770	LC348453	LC348663	Kondo & Shimai 5776-LPCGS (HIRO)
***P*. *medusina***	AB199771	LC348454	LC348664	Kondo & Shimai 710-LPCGS (HIRO)
***P*. *mesophytica***	AB251604	n/a	n/a	Kondo & Shimai 5777-LPCGS (HIRO)
***P*. *mirandae***	AB251605	LC348455	LC348665	Kondo & Shimai 5778-LPCGS (HIRO)
***P*. *moctezumae***	AB199772	AF531797	LC348666	Kondo & Shimai 5779-LPCGS (HIRO)
***P*. *moranensis***	AB199773	AF531798	LC348667	Kondo & Shimai 5780-LPCGS (HIRO)
***P*. *mundi***	AB198354	AF531800	LC348668	Kondo & Shimai 5748-LPCGS (HIRO)
***P*. *nevadensis***	AB198355	DQ010664	LC348669	Kondo & Shimai 5749-LPCGS (HIRO)
***P*. *nivalis***	AB199774	LC348456	LC348670	Kondo & Shimai 5781-LPCGS (HIRO)
***P*. *oblongiloba***	AB199775	LC348457	LC348671	Kondo & Shimai 712-LPCGS (HIRO)
***P*. *parvifolia***	AB199777	n/a	n/a	Kondo & Shimai 713-LPCGS (HIRO)
***P*. *pilosa***	AB199778	n/a	LC348672	Kondo & Shimai 714-LPCGS (HIRO)
***P*. *planifolia***	AB212109	LC348458	LC348673	Kondo & Shimai 5800-LPCGS (HIRO)
***P*. *poldinii***	AB198356	AF531804	LC348674	Kondo & Shimai 702-LPCGS (HIRO)
***P*. *potosiensis***	AB199779	LC348459	LC348675	Kondo & Shimai 5782-LPCGS (HIRO)
***P*. *primuliflora***	AB212110	DQ010666	LC348676	Kondo & Shimai 5801-LPCGS (HIRO)
***P*. *pumila***	AB212111	LC348460	LC348677	Kondo & Shimai 5802-LPCGS (HIRO)
***P*. *ramosa***	AB198357	DQ010667	LC348678	Kondo & Shimai 5735-LPCGS (HIRO)
***P*. *rectifolia***	AB199780	AF531801	n/a	Kondo & Shimai 5783-LPCGS (HIRO)
***P*. *reticulata***	AB199781	LC348451	n/a	Kondo & Shimai 5784-LPCGS (HIRO)
***P*. *rotundiflora***	AB199782	AF531802	LC348679	Kondo & Shimai 5785-LPCGS (HIRO)
***P*. *sharpii***	AB199783	AF531803	LC348680	Kondo & Shimai 5786-LPCGS (HIRO)
***P*. *vallisneriifolia***	AB198358	AF531805	LC348681	Kondo & Shimai 5750-LPCGS (HIRO)
***P*. *vallis-regiae***	n/a	n/a	LC348682	Kondo & Shimai 719-LPCGS (HIRO)
***P*. *variegate***	AB198359	DQ010668	LC348683	Kondo & Shimai 5736-LPCGS (HIRO)
***P*. *villosa***	AB198360	DQ010669	LC348684	Kondo & Shimai 5737-LPCGS (HIRO)
***P*. *vulgaris***	AB198361	AF531806	LC348685	Kondo & Shimai 5751-LPCGS (HIRO)
***P*. *zecheri***	AB199784	LC348461	LC348686	Kondo & Shimai 5787-LPCGS (HIRO)
***Genlisea hispidula***	AB212112	n/a	LC348687	Kondo & Shimai 704-LPCGS (HIRO)
***G*. *lobata***	AB212113	n/a	LC348688	Kondo & Shimai 705-LPCGS (HIRO)
***G*. *pallida***	AB212114	n/a	LC348689	Kondo & Shimai 706-LPCGS (HIRO)
***G*. *repens***	AB212115	n/a	LC348690	Kondo & Shimai 707-LPCGS (HIRO)
***G*. *violacea***	AB212116	n/a	LC348691	Kondo & Shimai 703-LPCGS (HIRO)
***Utricularia alpine***	AB212117	AF531822	LC348692	Kondo & Shimai 708-LPCGS (HIRO)
***U*. *floridana***	n/a	n/a	LC348693	Whitten s.n. (FLAS)
***U*. *gibba***	n/a	n/a	LC348694	Shimai s.n. (TNS)
***U*. *minor***	AB212118	n/a	n/a	Kondo & Shimai 5755-LPCGS (HIRO)

Sampled taxa are listed in alphabetical order. Sequence data are available from the International Nucleotide Sequence Database (INSD; http://www.insdc.org/). Herbarium codes: FLAS = Florida Museum of Natural History; HIRO = Hiroshima University; TNS = National Museum of Nature and Science. n/a = sequence data not available.

### DNA extraction

#### From fresh leaves

After washing the fresh leaves, water was removed completely using Kimwipes (Nippon Paper Crecia Co., Tokyo, Japan) and the leaves were kept at −60°C in an ultra-low temperature freezer. The frozen fresh leaf for each sample (0.07–0.1 g per sample) was finely ground in liquid nitrogen. DNA isolation from the ground samples was carried out using the ISOPLANT II (Nippon Gene, Tokyo, Japan) kit following the manufacturer’s protocol.

#### From dried leaves

Dust and insects stuck on the dried leaves were carefully removed using cotton buds moistened with 70% ethanol. The dried leaf (0.020–0.025 g per sample) was finely ground in liquid nitrogen. Isolation of DNA from the ground samples was carried out using the DNeasy® Plant Mini Kit (Quiagen, Hilden, Germany) following the manufacturer’s protocol.

### Amplification of DNA

#### ITS

The DNA sample was amplified by polymerase chain reaction (PCR) using TaKaRa *LA Taq*^TM^ (Takara Bio Inc., Kusatsu, Japan) with GC buffer II, included in the kit. The forward primer was 20 pmol/μL of ITS5 and the reverse primer was 20 pmol/μL of ITS4 [45]. The samples were incubated for an initial 2 min at 94°C and then 33 cycles of 50 s denaturation at 94°C, 1 min annealing at 48°C and 30 s extension at 72°C. When the amplification was insufficient, 20 pmol/μL of AB101 for forward and AB102 primers for reverse [46] were used instead of ITS5 and ITS4 primers. The samples were incubated for an initial 2 min at 94°C and then 33 cycles of 50 s denaturation at 94°C, 1 min annealing at 60°C, and 30 s extension at 72°C. The PCR products were then purified from collected agarose gels containing the targeted DNA region using the GFX^TM^ PCR DNA and Gel Band Purification Kit (Amersham Biosciences, Piscataway, New Jersey, USA) following the manufacturer’s protocol. For cycle sequencing, the samples were incubated for an initial 1 min at 96°C, and then 35 cycles of 10 s denaturation at 96°C, 5 s annealing at 50°C, and 80 s extension at 72°C.

#### matK

The basic protocol used was that mentioned in Cieslak *et al*. [32] and primer sets used were identical with those in Cieslak *et al*. [32] and Beck *et al*. [35]. One forward primer “Ping_trnK-F2 (5’–TCC CCT CCA TCA GGG GAT TCT–3’)” was designed in this study. Apart from the sequence data (39 taxa) from Cieslak *et al*. [32] and Beck *et al*. [35], additional DNAs from 30 taxa were amplified at Kyoto University to add to this study.

#### rpl32-trnL

The region was amplified using Phusion Green Hot Start II High-Fidelity DNA Polymerase (Thermo Scientific, Waltham, Massachusetts, USA) with 0.6 μL of DMSO per sample following the manufacturer’s protocol at the Florida Museum of Natural History, University of Florida. The primers used were *rpL32*–F for forward and *trnL*^(UAG)^ for reverse [47]. The samples were incubated for an initial 45 s at 98°C and then 32 cycles of 10 s denaturation at 98°C, 30 s annealing at 55°C, and 40 s extension at 72°C. Finally, the samples were kept at 72°C for 5 min.

### Phylogenetic analyses

The DNA sequence matrix was aligned by Genetyx-Win Version 5.2 (Software Development Co., Tokyo, Japan) using ‘Multiple Alignment’ function and was then adjusted manually. The sequence data are available from the INSD under the accession numbers summarised in Table 1.

Maximum likelihood (ML) analyses for each individual gene alignment were conducted using RAxML ver. 8.1.12 [48], with 1,000 replicates under the GTRGAMMA model since the best fit partition schemes identified by PartitionFinder [49] for all the datasets were equivalent (nst = 6, rates = gamma); all these analyses were implemented on the HiPerGator 2.0 at the University of Florida. *Genlisea* and *Utricularia* were selected as an outgroup. A tree from combined cpDNA datasets, *matK* + *rpl32-trnL*, was employed. All the trees were manipulated by MEGA [50] and R package phytools v0.7–00 [51].

For the Neighbor-Net analysis, each of the aligned three DNA datasets including the outgroup as done for the phylogenetic trees was imported to SplitsTree4 (Version 4. 14. 6; www.splitstree.org), and an unrooted phylogenetic network was constructed following the manual supplied by Hall [52]. The analysis was performed using the Neighbor-Net algorithm [53], loosely based on the Neighbor-Joining algorithm, to present complex evolutional pathways and reticulate relationships among the sampled taxa [54].

### Geographical distributions

The distribution area of the genus was divided into nine geographical regions based on the distribution ranges of taxa and geographical barriers: CAM = Central America (Guatemala to Panama); CRB = the Caribbean (the Bahamas, Cuba, and Hispaniola); EUR = Europe (west of the Urals, including the British Isles, and Iceland); MEX = Mexico; NAF = North Africa (Morocco); NAM = North America (Canada, USA, the Aleutians, Greenland, but excluding Mexico); NAS = Northeastern Asia (east of the Urals, Siberia, the Russian Far East, Kamchatka, Sakhalin, the Kuril Islands, Mongolia, China, the Himalayas, and Japan); SAM = South America (from Venezuela to Tierra del Fuego through the Andes and Patagonia); WAS = Western Asia (Cyprus, Anatolia, and the Caucasus). The geographical distribution of each taxon sampled is presented in Table 2. Only a few species are ubiquitously distributed in the area, while many others occur in a single country or on a specific mountain, or island. Taxa which form hibernacula are found in the temperate regions or higher elevations of EUR, NAF, NAM, NAS, and WAS, and those geographical regions are treated as the temperate Northern Hemisphere in this article. A few species are distributed in both Mexico and Central America while most species are endemic to Mexico, and thus the species are treated as the Mexican group unless necessary to distinguish.

**Table 2:** Geographical distributions of *Pinguicula* taxa examined in this study.

Taxon	Subg	Distribution area by geographical region								
		CAM	CRB	EUR	MEX	NAF	NAM	NAS	SAM	WAS
***Pinguicula acuminata* Benth.**	Iso				✓					
***P*. *agnata* Casper**	Iso				✓					
***P*. *albida* C.Wright ex Griseb.**	Iso		✓							
***P*. *alpina* L.**	Tem			✓				✓		
***P*. *antarctica* Vahl**	Tem								✓	
***P*. *balcanica* subsp. *balcanica* Casper**	Pin			✓						
***P*. *balcanica* subsp. *pontica* Casper**	Pin									✓
***P*. *benedicta* Barnhart**	Iso		✓							
***P*. *bissei* Casper**	Iso		✓							
***P*. *bohemica* Krajina**	Pin			✓						
***P*. *caerulea* Walter**	Iso						✓			
***P*. *calyptrata* Kunth**	Tem								✓	
***P*. *chilensis* Clos**	Tem								✓	
***P*. *chuquisacenis* S.Beck, A.Fleischm. & Borsch**	Tem								✓	
***P*. *colimensis* McVaugh & Mickel**	Pin				✓					
***P*. *conzattii* Zamudio & van Marm**	Iso				✓					
***P*. *corsica* Bernard & Gren. ex Gren. & Godr.**	Pin			✓						
***P*. *crassifolia* Zamudio**	Pin				✓					
***P*. *crenatiloba* DC.**	Tem	✓			✓					
***P*. *crystallina* Sm.**	Iso									✓
***P*. *cubensis* Urquiola & Casper**	Iso		✓							
***P*. *cyclosecta* Casper**	Pin				✓					
***P*. *debbertiana* Speta & F.Fuchs**	Pin				✓					
***P*. *dertosensis* (Cañig.) Mateo & M.B.Crespo**	Pin			✓						
***P*. *ehlersiae* Speta & Fuchs**	Pin				✓					
***P*. *elongata* Benj.**	Tem								✓	
***P*. *emarginata* Zamudio & Rzed.**	Tem				✓					
***P*. *esseriana* B.Kirchner**	Pin				✓					
***P*. *filifolia* C.Wright ex Griseb.**	Iso		✓							
***P*. *fiorii* Tammaro & Pace**	Pin			✓						
***P*. *gigantea* Luhrs**	Iso				✓					
***P*. *gracilis* Zamudio**	Tem				✓					
***P*. *grandiflora* Lam.**	Pin			✓		✓				
***P*. *gypsicola* Brandegee**	Pin				✓					
***P*. *hemiepiphytica* Zamudio & Rzed.**	Pin				✓					
***P*. *heterophylla* Benth.**	Iso				✓					
***P*. *hirtiflora* Ten.**	Iso			✓						
***P*. *ibarrae* Zamudio**	Iso				✓					
***P*. *immaculata* Zamudio & Lux**	Tem				✓					
***P*. *involuta* Ruiz & Pav.**	Tem								✓	
***P*. *ionantha* R.K.Godfrey**	Iso						✓			
***P*. *jackii* var. *jackii* Barnhart**	Pin		✓							
***P*. *jackii* var. *parviflora* Ernst**	Pin		✓							
***P*. *jaumavensis* Debbert**	Pin				✓					
***P*. *laueana* Speta & F.Fuchs**	Pin				✓					
***P*. *leptoceras* Rchb.**	Pin			✓						
***P*. *lignicola* Barnhart**	Iso		✓							
***P*. *lilacina* Schltdl. & Cham.**	Iso	✓			✓					
***P*. *longifolia* subsp. *caussensis* Casper**	Pin			✓						
***P*. *longifolia* subsp. *longifolia* Ramond ex DC.**	Pin			✓						
***P*. *longifolia* subsp. *reichenbachiana* (Schindler) Casper**	Pin			✓						
***P*. *lusitanica* L.**	Iso			✓		✓				
***P*. *lutea* Walter**	Iso						✓			
***P*. *macroceras* Link**	Pin						✓	✓		
***P*. *macrophylla* Kunth**	Pin				✓					
***P*. *medusina* Zamudio & Studnička**	Iso				✓					
***P*. *mesophytica* Zamudio**	Pin	✓			✓					
***P*. *mirandae* Zamudio & Salinas**	Iso				✓					
***P*. *moctezumae* Zamudio & R.Z.Ortega**	Pin				✓					
***P*. *moranensis* Kunth**	Pin	✓			✓					
***P*. *mundi* Blanca, Jamilena, Ruíz Rejón & Reg.Zamora**	Pin			✓						
***P*. *nevadensis* (H.Lindb.) Casper**	Pin			✓						
***P*. *nivalis* Luhrs & Lampard**	Tem				✓					
***P*. *oblongiloba* DC.**	Pin				✓					
***P*. *parvifolia* B.L.Rob.**	Iso				✓					
***P*. *pilosa* Luhrs, Studnička & Gluch**	Iso				✓					
***P*. *planifolia* Chapm.**	Iso						✓			
***P*. *poldinii* J.Steiger & Casper**	Pin			✓						
***P*. *potosiensis* Speta & F.Fuchs**	Pin				✓					
***P*. *primuliflora* C.E.Wood & R.K.Godfrey**	Iso						✓			
***P*. *pumila* Michx.**	Iso		✓				✓			
***P*. *ramosa* Miyoshi**	Tem							✓		
***P*. *rectifolia* Speta & F.Fuchs**	Pin				✓					
***P*. *reticulata* Schlauer**	Iso				✓					
***P*. *rotundiflora* Studnička**	Iso				✓					
***P*. *sharpii* Casper & K.Kondo**	Iso				✓					
***P*. *vallisneriifolia* Webb**	Pin			✓						
***P*. *vallis-regiae* F.Conti & Peruzzi**	Pin			✓						
***P*. *variegata* Turcz.**	Tem							✓		
***P*. *villosa* L.**	Pin			✓			✓	✓		
***P*. *vulgaris* L.**	Pin			✓		✓	✓	✓		✓
***P*. *zecheri* Speta & F.Fuchs**	Pin				✓					

Subgeneric division *sensu* Casper: Iso = subgenus *Isoloba*; Pin = subgenus *Pinguicula*; Tem = subgenus *Temnoceras*. Distribution area: CAM = Central America; CRB = the Caribbean; EUR = Europe; MEX = Mexico; NAF = North Africa; NAM = North America; NAS = Northeastern Asia; SAM = South America; WAS = Western Asia.

## Results

### Phylogenetic trees

#### ITS

**Fig 3:**
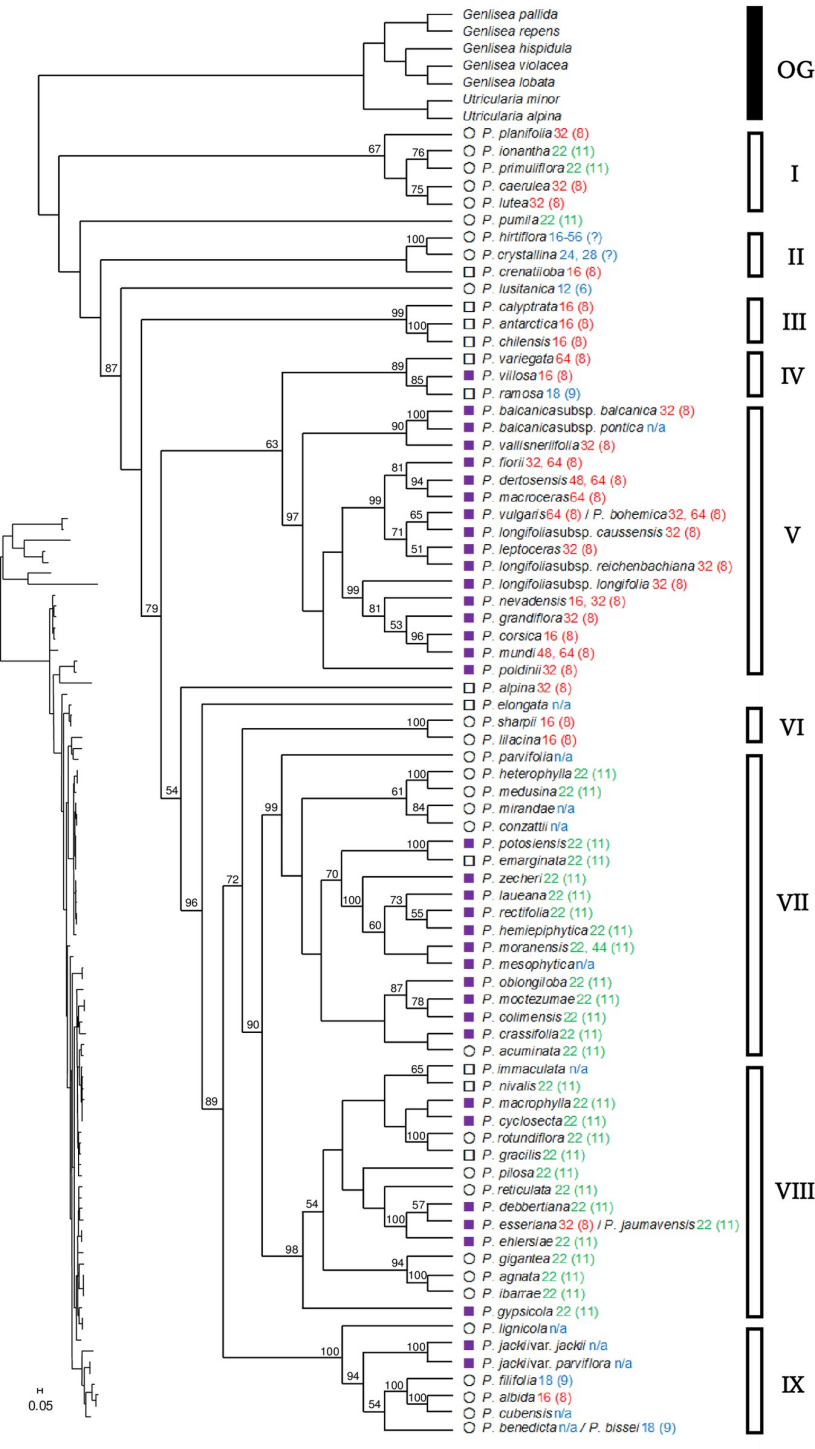
Phylogenetic tree of *Pinguicula* taxa from ITS inferred by RAxML. The numbers above branches show bootstrap supports (%), but those of 50% or less and those for the outgroup are not shown. Three subgenera *sensu* Casper are shown as open circles for *Isoloba*, purple squares for *Pinguicula*, and open squares for *Temnoceras*. The number after scientific name and that in brackets are chromosome number and basic chromosome number, respectively; the basic chromosome number of *x* = 8 and *x* = 11 are coloured in red and green, respectively, and other numbers or unreported (n/a) are in blue. OG and Roman numerals indicate the outgroup and major clade numbers, respectively.

The length of ITS-1 and ITS-2 was between 573 and 717 base pairs (bp). The informative site was 601 in the aligned length of 981 bp. The ITS tree could be divided into nine major clades although some bootstrap supports (BS), particularly near the base of the tree, were weak (Fig 3). Roman numerals in the figure indicate major clade numbers. Clade I (67% BS) consisted of five species from the southeastern USA. Clade II (< 50% BS) consisted of *P*. *crystallina* Sm. and *P*. *hirtiflora* Ten. from the Mediterranean Basin and *P*. *crenatiloba* DC. from Mexico and Central America. Clade III (99% BS) consisted of South American *P*. *antarctica* Vahl, *P*. *calyptrata* Kunth, and *P*. *chilensis* Clos. Clade IV (89% BS) consisted of three small-rosetted species (< 30 mm in rosette diameter), *P*. *ramosa* Miyoshi, *P*. *variegata* Turcz., and *P*. *villosa* L., of which the former two were restricted to Eastern Eurasia while *P*. *villosa* was very widely scattered in the boreal regions of Eurasia and North America. Clade V (97% BS) consisted of 17 taxa (e.g., *P*. *grandiflora* Lam., *P*. *vulgaris* L., etc.) which were found in the temperate Northern Hemisphere. All the taxa in Clades IV and V form rootless hibernacula. Clade VI (100% BS) consisted of only two morphologically very similar, if not identical, homophyllous species *P*. *lilacina* Schltdl. & Cham. and *P*. *sharpii* Casper & K.Kondo. The former is very widely but sparsely distributed in Mexico and Central America while the latter is endemic to Chiapas, Mexico. Clade VI is closely related to Clades VII and VIII. Clade VII (99% BS) with 18 species is mostly found in the mountain ranges of western Mexico to Central America, with the exception of *P*. *moranensis* Kunth, which exhibits a much wider distribution range than the others. Clade VIII (98% BS) consisted of 16 species mostly found in the mountain ranges of eastern Mexico. The species in Clades VII and VIII characteristically form winter rosettes. Clade IX (100% BS) consisted of seven Cuban taxa. *Pinguicula pumila* Michx., distributed in the southeastern USA and the Bahamas, did not form a clade with other species, but it is related to Clade I. The other three species, *P*. *lusitanica* L., *P*. *alpina* L., and *P*. *elongata* Benj. did not belong to any of the major clades mentioned above.

#### Concatenated cpDNA

**Fig 4:**
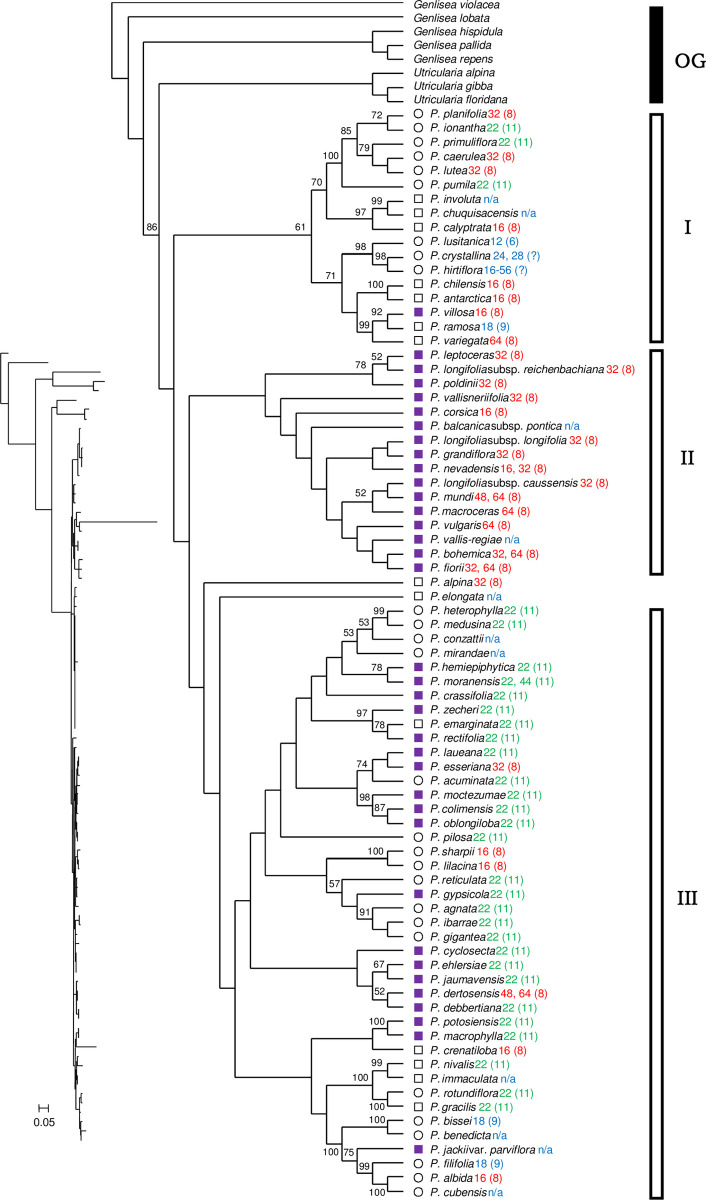
Phylogenetic tree of *Pinguicula* taxa from concatenated cpDNA inferred by RAxML. See Fig 3 for figure legends.

The concatenated cpDNA (*matK* + *rpl32-trnL*) tree could be divided into at least three major clades (Fig 4). Clade I (61% BS) consisted of 17 species which are from various geographical regions, such as the southeastern USA, South America, the Mediterranean Basin, or the boreal region of the Northern Hemisphere. Clade II (< 50% BS) consisted of 16 taxa, all of which form rootless hibernacula, from the temperate Northern Hemisphere. Clade III (< 50% BS) consisted of 42 taxa from Mexico, Central America, and Cuba, except *P*. *dertosensis* (Cañig.) Mateo & M.B.Crespo from Spain. Clade III can be divided into several subclades. Two species, *P*. *alpina* and *P*. *elongata*, did not belong to any of the major clades mentioned above. All the clades had low BS (< 50%) at the base of the tree.

#### matK

The length of *matK* sequence was approximately 2,500 bp, although there were some incomplete sequence data available from the INSD. The informative site was 342 in the aligned total length of 2,674 bp. The *matK* tree could be divided into at least three major clades (S1 Fig). Clade I (98% BS) consisted of 39 taxa from Mexico, Central America, or Cuba, but with the exception of *P*. *dertosensis* (Cañig.) Mateo & M.B.Crespo from Spain. The clade could further be divided into a number of subclades. Clade II (95% BS) consisted of 14 taxa from the temperate Northern Hemisphere. All the 14 taxa in Clade II which form rootless hibernacula were the most well-differentiated group in this analysis. Clade III (< 50% BS) with 14 species was rather a miscellaneous group in terms of the biogeography and could be divided into a few subclades. This clade contained the three small-rosetted species from the Northern Hemisphere, homophyllous *P*. *hirtiflora* and *P*. *lusitanica* from Europe, and species from the southeastern USA and South America. Two species, *P*. *alpina* and *P*. *elongata*, did not belong to any of the major clades mentioned above.

#### rpl32-trnL

The total length of sequence including *rpl32-trnL* was between 504 and 695 bp. The informative site was 361 in the aligned sequence length of 1,109 bp. The *rpl32-trnL* tree consists of four major clades (S2 Fig). A number of low BS (< 50%) were found on the tree. Clade I (81% BS) consisted of 18 taxa, all of which forming hibernacula are from the temperate Northern Hemisphere. Clade II (75% BS) was a geographically miscellaneous group that consisted of 11 taxa from Europe, Anatolia, the southeastern USA, or South America. Clade III (< 50% BS), which could be divided into three or four subclades, consisted of 31 species from Mexico, except South American *P*. *elongata*. Clade IV (< 50% BS) consisted of six Cuban taxa. Three small-rosetted species, *P*. *ramosa*, *P*. *variegata*, and *P*. *villosa*, did not belong to any of the major clades mentioned above.

#### Incongruence between phylogenetic trees

**Fig 5:**
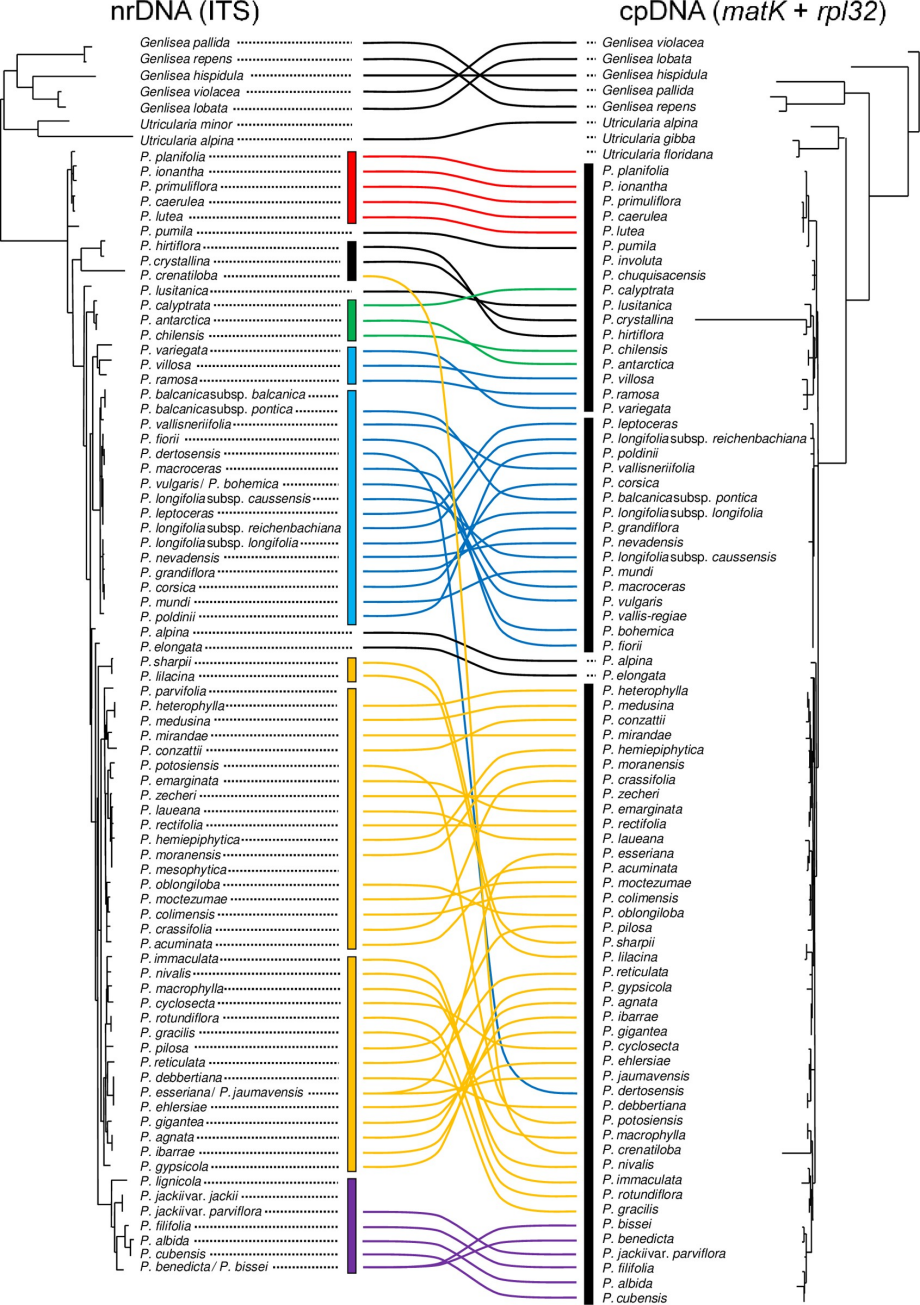
Phylogenetic comparison of nrDNA (ITS) and concatenated cpDNA. The figure shows topological incongruence between the ITS and combined cpDNA (*matK* + *rpl32-trnL*) trees. Vertical bars and connected lines are coloured based on major clades in the ITS tree; red for Clade I (the southeastern USA), green for Clade III (South America), blue for Clades IV and V (the temperate Northern Hemisphere), gold for Clades VI, VII, and VIII (Mexico and Central America), purple for Clade IX (Cuba), and black for others and the outgroup.

Incongruence was apparent between the nrDNA and combined cpDNA trees as shown in Fig 5, which illustrates topological differences. The branching order and the number of clades were inconsistent between the trees. Taxa from the temperate Northern Hemisphere which form rootless hibernacula were the most well-differentiated lineage in each tree. Species from Mexico which form winter rosettes showed a similar tendency, although those species and Cuban taxa appeared in the same clade in the combined cpDNA tree. Such incongruence was also seen among the trees based on the individual markers.

### Phylogenetic networks

#### ITS

**Fig 6:**
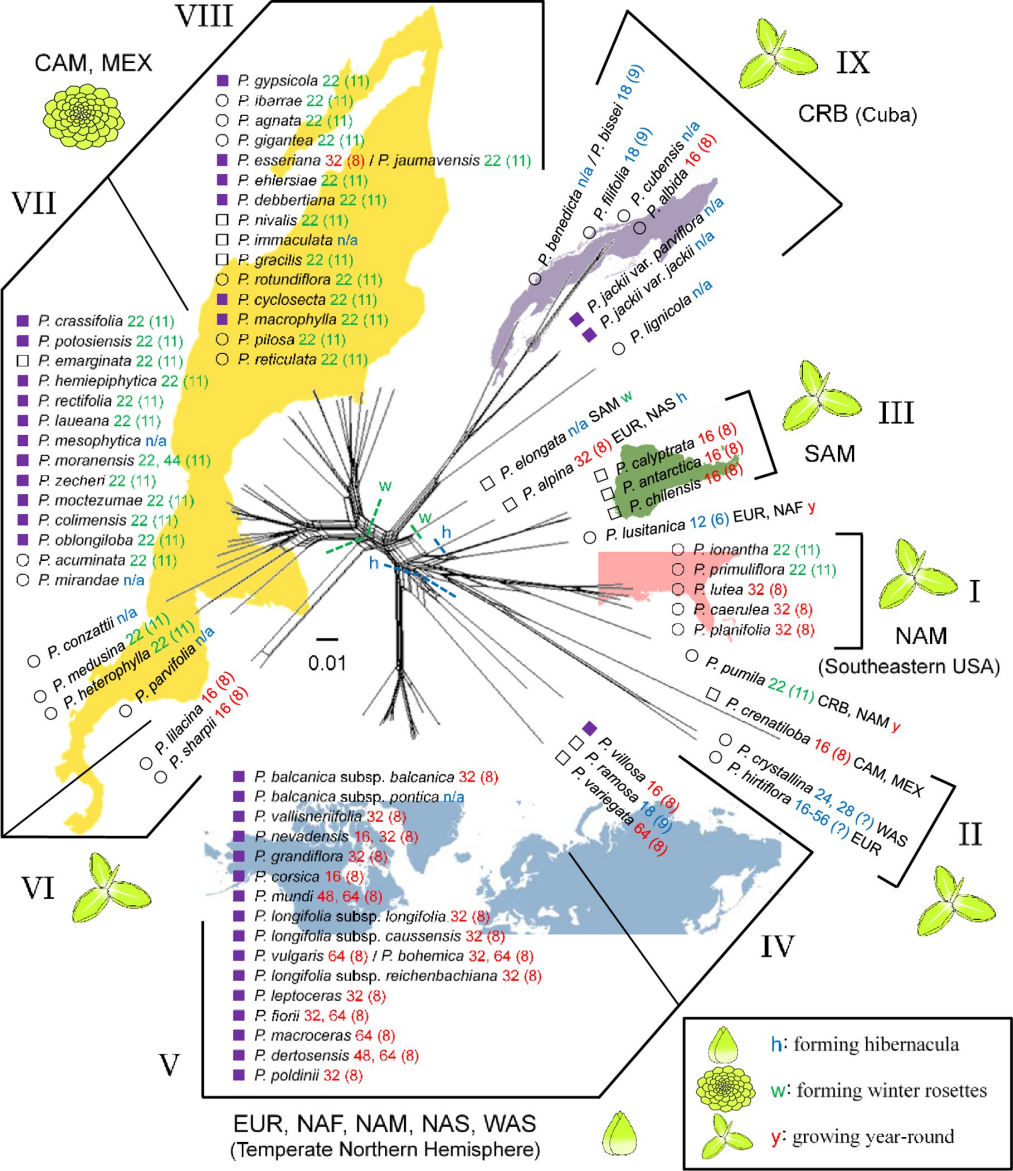
Phylogenetic network of *Pinguicula* taxa from ITS inferred by Neighbor-Net analysis. Three subgenera *sensu* Casper are shown as open circles for *Isoloba*, purple squares for *Pinguicula*, and open squares for *Temnoceras*. Abbreviations for the geographical distribution area are listed in the Materials and Methods section. The number after the scientific name and that in brackets are chromosome number and basic chromosome number, respectively; the basic chromosome number of *x* = 8 and *x* = 11 are coloured in red and green, respectively, and other numbers or unreported (n/a) are in blue. The outgroup is not shown in this figure but is included for the analysis. Roman numerals indicate major clade numbers shown in the phylogenetic tree from the same DNA region. Broken lines with “h” and “w” represent hypothetical acquirement of hibernaculum formation and that of winter rosette formation, respectively. The map image was made with Natural Earth (https://www.naturalearthdata.com/).

The ITS phylogenetic network accorded with the ITS phylogenetic tree. The edge groups in the network (Fig 6) and major clades in the tree are basically consistent. However, *P*. *crenatiloba* divided from the edge group of *P*. *crystallina* and *P*. *hirtiflora*, all of which were in the same clade in the ITS tree. The edge groups largely corresponded with geographical distributions, basic chromosome numbers (haploidy), and life-forms, but were inconsistent with the three subgenera *sensu* Casper. Reticulation events, identified as parallel edges in the network, among the ancestors of the edge groups were active in this DNA region, suggesting ancient gene flow or introgression.

#### matK

The *matK* phylogenetic network (S3 Fig) also accorded with the *matK* phylogenetic tree. The edge groups contain miscellaneous taxa in terms of geographical distributions, life-forms, and basic chromosome numbers as well as the three-subgeneric division except the edge group containing the taxa from the temperate Northern Hemisphere. Reticulation events among the ancestors of the edge groups were suggested to be inactive in this DNA region.

#### rpl32-trnL

Similarly, the *rpl32-trnL* phylogenetic network (S4 Fig) accorded with the *rpl32-trnL* phylogenetic tree. The edge groups and major clades largely corresponded, although the edges of *P*. *lusitanica* and *P*. *crystallina* were somewhat independent within the edge group. Only two edge groups from the temperate Northern Hemisphere and Cuba were well-differentiated in terms of geographical distributions and life-forms. Reticulation events among the ancestors of the edge groups were active in this DNA region.

## Discussion

### Phylogenetic analyses

#### ITS

The ITS tree and network are well-supported by the biogeographical patterns of the genus *Pinguicula* as well as life-forms and basic chromosome numbers (Figs 3 and 6). The results give strength to the hypothesis that a specific lineage acquired the same life-form in a geographical region. The network suggests that gene flow in nrDNA had been extensive among ancestral taxa of the genus prior to their geographical isolation. Low BS at the base of the tree can be attributed to complex reticulation events in the early evolutionary history [55], although each major clade in the tree has higher BS. After geographical and genetic isolation of the ancestral taxa by changes in climate, rapid speciation took place in association with migration. The short branch length on the tree represents rapid speciation in each lineage and a number of species seen today are rather modern. In Mexico, for example, considerable morphological diversity among species is seen (Fig 2); however, they have emerged from a common ancestor in a short time span and are phylogenetically close relatives. The results suggest that the common ancestor of Clades VII and VIII in Mexico acquired the formation of winter rosettes before extensive speciation. Similarly, that of Clades IV and V in the temperate Northern Hemisphere acquired the formation of rootless hibernacula (Fig 6). Therefore, dormant strategies in the two lineages are different evolutionary modes.

#### Concatenated cpDNA

Regarding the concatenated cpDNA markers (*matK* + *rpl32-trnL*), the topology shows no clear correspondence with the morphology-based classification, physiological character, or geographical distribution (Fig 4), suggesting that the result is inconclusive. Topological incongruence is clearly seen between nrDNA (ITS) and cpDNA (*matK* + *rpl32-trnL*) (Fig 5). It would be better to discuss *matK* and *rpl32-trnL* individually rather than the combined cpDNA dataset.

#### matK

In this DNA region, presenting larger clades, higher BS (> 89% BS) is seen at the base of clades in the tree (S1 Fig). The network suggests relatively infrequent reticulation events among the ancestral taxa (S3 Fig). The results indicate that taxa in Clade II from the temperate Northern Hemisphere form a well-differentiated group. It is unclear why Spanish *P*. *dertosensis* appears in the Mexican group, but this could be for several possible reasons (see [53, 56]), of which the most plausible is higher homology in the DNA region between taxa. Alternatively, some incomplete sequence data available from the INSD may have affected the analysis. It could be interpreted, although this is disputable, that the genus acquired the dormant strategy in the early evolutionary stage, but it was then lost in some lineages, as also suggested by Beck *et al*. [35]. The results here showed that the three small-rosetted species (*P*. *ramosa*, *P*. *variegata*, and *P*. *villosa*) were related to species from the southeastern USA and South America, which differ from the results in Beck *et al*. [35], showing the three were more closely related to Mexican species. The topological difference between the two *matK* trees can be attributed to the number of samples used.

#### rpl32-trnL

Although it is not as clear as in the ITS tree, clades in the *rpl32-trnL* tree are partially geographically dependent (S2 Fig). In contrast to the *matK* tree, Mexican and Cuban taxa are different lineages. Clade II consists of geographically various taxa, which are from the Mediterranean Basin, the southeastern USA, or South America. The *rpl32-trnL* tree, in comparison to the ITS tree, seems to demonstrate the relationship between biogeographical patterns and life-forms less clearly. It does not, however, completely deny the hypothesis that the evolutionary history of the genus is associated with geographical distributions. More extensive ancient reticulation events are suggested by the network (S4 Fig), in contrast to the *matK* network, suggesting different modes of inheritance within the same organelle.

#### Incongruence between nrDNA and cpDNA

Low congruence and different branching orders are seen between nrDNA and cpDNA (Fig 5). Such incongruence is not uncommon [57–60]. In angiosperms, more than 80% are maternal inheritance in cpDNA [61]; however, lateral gene transfer or gene capture has also been reported [62]. Namely, it is possible that different inheritances in nrDNA and cpDNA linked to introgressive hybridisation resulted in incongruence between the DNA regions. Even in the same organelle, topological discrepancies in the phylogenetic trees between *matK* and *rpl32-trnL* are clearly seen. Some factors, such as genetic heterogeneity, genetic polymorphism, or incomplete lineage sorting, may cause discrepancies among phylogenetic trees [53, 56, 62]. Topological incongruence among the DNA datasets suggests complex gene flows.

### Life-forms

At least three life-forms of *Pinguicula* can be distinguished; 1) forming winter rosettes to resist a dry winter, 2) forming hibernacula to survive during a frigid winter, and 3) growing throughout the year. Based on the *matK* analysis, Beck *et al*. [35] hypothesised that hibernaculum formation evolved only once, but some species subsequently lost the dormant strategy or transformed into winter rosette formation in the section *Temnoceras sensu* Fleischmann & Roccia, which includes Mexican and Cuban taxa. The results obtained from ITS, on the other hand, suggest that the formations of winter rosette and hibernaculum are different synapomorphies that have arisen independently in different lineages and geographical regions for adaptation to local climates. However, Eurasian *P*. *alpina*, which form rooted hibernacula, and South American *P*. *elongata*, which form ovoid winter rosettes (resembling rooted hibernacula), are exceptions having the dormant strategy as a result of parallel evolution (Fig 6). In Mexico and Europe, both year-round growth and dormant species are occasionally seen sympatrically within a microhabitat, but the latter species are more specialised and advantageous for winter survival.

### Floral morphology

Floral morphology in the genus is still believed to be an important characteristic for the classification and identification, but the similarity of flowers between allopatric geographical regions is more likely as a result of convergent evolution according to the results obtained in this study. For example, Casper [3] placed *P*. *vulgaris*, from the temperate Northern Hemisphere, and *P*. *moranensis*, from Mexico and Guatemala, both having zygomorphic purple flowers, into the subgenus *Pinguicula*; however, none of the present results support his treatment as they are phylogenetically different lineages. The corolla tube continuing into a nectar spur in taxa from the temperate Northern Hemisphere is dorsally compressed, but that from Mexico is often not. Mexican species exhibiting floral diversity are divided into the three subgenera; however, the results suggest that they are monophyletic, except *P*. *crenatiloba*.

### Evolutionary history

Both major clades in the tree and edge groups in the network based on ITS accord well with life-forms. All the taxa in Clades IV and V from the temperate Northern Hemisphere form rootless hibernacula, and all the species in Clades VII and VIII from Mexico form winter rosettes (exceptionally, *P*. *emarginata* Zamudio & Rzed. and *P*. *moctezumae* Zamudio & R.Z.Ortega form winter rosettes only under a severe dry conditions, and *P*. *gigantea* Luhrs does not form a conspicuous winter rosette). Taxa in the remaining major clades grow year-round, although note that some may form smaller rosettes with shorter leaves or reduce their growth rate in winter but maintain the summer rosette form. It could be interpreted that the formation of winter rosettes or hibernacula is not a result of convergent evolution among different subgenera from multi-ancestors, but it is, according to the results, evaluated to be a phylogenetic constraint within a lineage (as stated, *P*. *alpina* and *P*. *elongata* being the exception). Such a genetically closely related group acquired a winter dormant strategy as a morphological adaptation to a local climate, but the rest of species remain homophyllous. Taxa forming hibernacula spread to cooler regions and higher mountains of the Northern Hemisphere, and those forming winter rosettes spread to Mexico.

In contrast to ITS, incomplete lineages in cpDNA caused by hybridisation and/or introgression do not always allow us to trace their phylogenetic relationships. The results of cpDNA, concerning biogeographical patterns and other traits, are ambiguous and are not fully explainable. Soltis *et al*. [62] reported that if chloroplast capture via hybridisation was involved in speciation, which commonly occurred in angiosperms, phylogenetic constructions using cpDNA could not resolve relationships within a taxonomic group. Even if a foreign chloroplast capture through introgressive hybridisation is evident, a nuclear genome may have been retained [62]. Fior *et al*. [63] stated that ITS was potentially more precise than *matK*. As stated, ITS was more informative than the other datasets due to higher substitutions. Therefore, further discussions focus mainly on the results of ITS.

According to the present ITS results, all the species in Clades VI, VII, and VIII are confined to Mexico except for a few that extend farther south into Central America. In Clade VI, *P*. *lilacina* is sparsely widespread from Mexico to Central America while *P*. *sharpii* is known only from the type locality in the state of Chiapas, Mexico. Both are annual to short-lived perennial homophyllous species. Eighteen species in Clade VII are mostly found in the Sierra Madre Occidental in Mexico to Central America through the Sierra Madre del Sur, with the exception of *P*. *moranensis*, which extends to the Sierra Madre Oriental and farther north to the state of Tamaulipas [64]. Sixteen species in Clade VIII are mostly found in the Sierra Madre Oriental, although a major conjunction of Clades VII and VIII is seen in the Central Mexican Plateau. Many of the species in Clades VII and VIII are confined to small geographical areas as micro-endemics often at higher elevations, e.g., *P*. *crassifolia* Zamudio is endemic to El Chico (2,800–3,000 m) in the state of Hidalgo.

Seventeen taxa which form rootless hibernacula in Clade V are found in the temperate Northern Hemisphere. Only a few species, such as *P*. *macroceras* Link or *P*. *vulgaris*, are more widely distributed while some others are endemics. At lower latitudes, they are mostly found at higher elevations with a cool climate as relics [65–67]. For example, *P*. *alpina*, widespread in Eurasia but more commonly found in the Alpes, the Scandinavian Peninsula, and the Himalayas, is regarded as a glacial relic [65]. In Europe, higher elevations of the Mediterranean Basin surrounded by warm and semiarid areas harbour more endemics than northern Europe. The three small-rosetted species, which form rootless hibernacula, in Clade IV, sister to Clade V, are more commonly found in north circumpolar regions or eastern Eurasia. Taxa in Clade V produce two or more scapes per year while the three species in Clade IV develop only a single scape. In addition, taxa in Clade V bear a few to numerous gemmae at the base of hibernacula for their vegetative reproduction, but those in Clade IV less frequently do so. Five species in Clade I and eight in Clade IX are endemic to the southeastern USA and Cuba, respectively.

Cuban and Mexican taxa are suggested to be a single group by the *matK* analysis. The ITS tree, however, shows that the two are phylogenetically differentiated groups, although both have arisen from a common ancestor. The ITS tree also suggests that *P*. *crenatiloba* from Mexico and Central America is more closely related to *P*. *crystallina* and *P*. *hirtiflora* from the Mediterranean region than the other Mexican species. The relationship between the two well-differentiated groups, European and Mexican, is unclear, but Eurasian *P*. *alpina* and South American *P*. *elongata* are related to the two groups.

The ITS results largely correspond with the basic chromosome numbers (haploidy) as well as geographical distributions (Fig 6). This suggests a correlation between the basic chromosome numbers and nrDNA evolution associated with hybridisation or subsequent speciation. It is known that allopolyploid hybridisation has a principal role in speciation of angiosperms [68–70]. Within a taxon, chromosome evolution generally increases the number of chromosomes or ploidy level which may subsequently result in morphological evolution [71–73] while reduction of the number is rather rare [74]. According to this theory, the higher basic chromosome number (*x* = 11) arose from the lower number. The taxa in the temperate Northern Hemisphere with the basic chromosome number of *x* = 8 multiplied the ploidy level in their evolutionary history. For example, *P*. *corsica* Bernard & Gren. ex Gren. & Godr. (2*n* = 16), endemic to higher elevations in Corsica, possesses the lowest ploidy level (diploid) in this group, whereas other taxa that spread across continental Europe are mostly polyploidy, e.g., tetraploid (2*n* = 4*x* = 32) or octoploid (2*n* = 8*x* = 64).

In Europe, hybrid speciation between *Pinguicula* taxa having different chromosome numbers could theoretically be possible in 16 × 48 = 32 (tetraploid) or 32 × 64 = 48 (hexaploid), although species with 2*n* = 48 are rare. Sympatric hybridisation between diploid species occasionally induces tetraploid offspring. Therefore, speciation in Europe involved the doubling of chromosome sets. Polyploid species are vigorous and potentially more adaptive to novel environments than diploid species [75]. In Mexico, on the other hand, chromosome evolution and morphological diversity (i.e., speciation) caused by increasing chromosome numbers cannot be explained since most species have 2*n* = 22. Thus, the basic chromosome number of *x* = 11 in Mexican species is a synapomorphic characteristic. The basic chromosome number in the species from the northeastern USA is either *x* = 8 or *x* = 11, and that in taxa from Cuba is either *x* = 8 or *x* = 9 [38], varying within each lineage. A further study on the cytology in those two groups will be needed to explain the variability or the possibility of parallel evolution in the basic chromosome numbers between lineages.

Although considerable morphological diversity among species, such as flower colour, leaf shape, or plant size, is seen at the local level, particularly in Mexico (Fig 2), the results suggest that they are basically monophyletic in a region. Similar examples have been reported in *Gaertnera* Lam. (Rubiaceae) [76] or taxa in Valerianaceae [77]. As presented in Lamiales including Lentibulariaceae by Müller *et al*. [8], much shorter branch lengths in *Pinguicula* were found on their *matK* tree, suggesting rapid speciation. The results presented here suggest that ancestral taxa migrated at least twice into Mexico and South America in their early evolutionary histories as there are two lineages in each of the regions, specifically the ancestors of *P*. *crenatiloba* and the other species in Mexico, and those of *P*. *elongata* and the other species in South America. The ITS results here suggest that South American *P*. *elongata* is phylogenetically related to Mexican species, but the other South American species are not. Ancestors of European taxa had more complex migrations.

No fossils of *Pinguicula* have hitherto been documented [32]; however, the divergence of *Pinguicula* and *Utricularia* was estimated to take place ca. 40 million years before present (yr BP) [78]. Which geographical region the genus originated remains still unspecified. *Pinguicula villosa*, widespread in *Sphagnum* bogs in the circumpolar and excessively cold regions of Eurasia and North America, is assumed to be an old species [19, 79], but it is not evident here. Although the expansion or intercontinental dispersal mechanism is unknown, land bridges in an ice age may be a possible explanation involving gene selection, fixation, and subsequent speciation [80]. It is plausible that ancestral taxa migrated through the land bridges in the north, but more evidence is needed to understand the dynamics of global dispersal of the genus. Rapid speciation occurred during or after the geographical isolation. Hybridisation and polyploidisation play important roles in generating rapid speciation [70]. Smith *et al*. [81] showed that nrDNA with biparental heredity in putative hybrids had higher coalescence than cpDNA. The phylogenetic incongruence among DNA regions found in the present study could be explained by introgression often caused by hybridisation among closely related parental taxa (e.g., [57–60, 80]).

Plant migration is often associated with changes in climate. In Mexico, altitudinal vegetation shifts caused by climatic changes are evident as it was cooler and wetter in the early Holocene [82, 83]. It is estimated that during the late glacial period (14,000–10,000 yr BP), the vegetation in Central Mexico descended at least 900 m, temperature was 5°C lower, and precipitation was 30% higher than today [84]. The temperature in Mexico started to rise rapidly ca. 10,000 yr BP, resulting in the plant distributions seen today [85]. Temperature and precipitation changes in the Holocene led to a decline in the plant population size. Some ancestral taxa might have been extinct due to habitat loss. With declining the population size, gene flow among ancestral taxa in neighboring populations occurred, and migration resulting in further isolation consequently accelerated genetic diversification [85]. However, vegetation shifts involving climate changes in the highlands of Central Mexico seem to be much more complex because of the geological structures associated with orogenies [82, 86]. A characteristic feature in the region is tephra deposits related to volcanic activities, affecting geological aspects [82].

Similar climatic conditions to those of Mexico existed in the Mediterranean Basin in the late Glacial to early Holocene period [87]. Divergence time within the genus is uncertain; however, the ancestral taxa might have been more widely distributed in the region. After a rise in temperature, the taxa remained in small patchy refugia at higher elevations or deep gorges surrounded by larger semiarid or warmer areas, often unfavourable for *Pinguicula*. In the Iberian Peninsula, as well as Mexico and other regions, *Pinguicula* is often found in alkaline calcareous soils, such as limestone or tufa, where other plant species are scarce. The complexity of mosaic landscapes, geographical variations, soil types, and cool climates at higher elevations resulted in their patchy distributions in specific ecological niches seen today, e.g., *P*. *vallisneriifolia* Webb in Andalusia (600–1,700 m), Spain. A few widespread species, such as *P*. *alpina*, *P*. *villosa*, or *P*. *vulgaris*, more commonly seen in the north, seem to have higher ecological adaptations to expand their distribution ranges.

It is expected that vicariance and allopatric parallel evolution by migration occurred within an incredibly short time span. Convergence and parallelism result in similar floral characteristics which sometimes mask and obscure phylogenetic relationships since such phenotypes are under rather simple genetic control [62]. Similar floral morphology in geographically separated regions is attributed to the convergence of pollination strategy associated with the local pollinator communities. Therefore, introgressive hybridisation which generated floral variations and subsequent gene selection involving bottleneck effects or founder effects could have promoted the floral diversity, or species richness, particularly in Mexico. Nonetheless, a further investigation of *P*. *moranensis*, which shows considerable morphological diversity, would be necessary to study whether it fits into a single species.

There is evidence supporting the idea of hybrid speciation in the genus. Interspecific natural hybrids (e.g., *P*. *grandiflora* × *P*. *vulgaris* = *P*. × *scullyi* Druce and *P*. *grandiflora* × *P*. *longifolia* subsp. *longifolia* Ramond ex DC.) have been reported from Europe [3–5]. Even though no apparent natural hybrids have been reported from Mexico, artificial hybrids can be easily produced through hand pollination among Mexican species [39]. Hybrids between Mexican species can further backcross or hybridise with other Mexican species and they are often fertile. This supports the hypothesis of rapid speciation with selection caused by introgressive hybridisation following geographical isolation (genetic drift). Indeed, some recently described species, particularly from Europe, resemble morphologically intermediates between species that have previously been described.

In some lineages, the chromosome numbers may be one of the clues to consider the evolutionary pathway of the genus. Allopolyploid speciation, multiplying the ploidy level, played a role in the temperate Northern Hemisphere (particularly in Europe). Some new species with increased ploidy levels are potentially more adaptive to vacant ecological niches and are able to expand their distributions [75]. On the other hand, homoploid hybridisation played a role and promoted species richness in Mexico.

A few widespread neospecies also crossed the land bridges before sea level rose to isolate their distribution areas. These species then became established in their new surroundings. For example, *P*. *grandiflora* migrated from the Iberian Peninsula or France to Ireland, but absent from the island of Great Britain, in the early postglacial age before sea levels were restored (the so-called Lusitanian floral elements) [5]. Another example is that *P*. *macroceras*, distributed in the northern Pacific regions, crossed the Bering Land Bridge (Beringia) and expanded its distribution range to Japan through the Kurils [88].

Major diversification of *Pinguicula* is particularly seen locally at higher elevations of semiarid areas, including Mexico and the Mediterranean Basin, where a dry climate often plays a role in the geographical isolation of species [21, 89]. Such unfavourable environmental barriers limit the availability of pollinators [70], which could have consequently promoted parallel floral evolution (or convergent floral evolution) among different geographical regions causing the morphological diversity within the genus. It is noteworthy that species richness of the genus, seen in small patchy refugia surrounded by unfavourable semiarid areas, was accelerated by environmental stress.

The results from the ITS sequence show that the major clades are basically geographically dependent. This is supported by life-forms and cytology. The genus *Pinguicula* is an example of a plant group in which floral morphology has masked and obscured phylogenetic relationships among species. It should be noted that the traditional classification, although it is important, is artificial. Those lineages presented by ITS in this study do not, therefore, fit to the three-subgeneric concept. In conclusion, we submit that the taxonomic revision of the genus *Pinguicula* based upon nrDNA is necessary.

## Supporting information

S1 Appendix List of synonymous manes. (DOCX)

S1 Fig Phylogenetic tree of *Pinguicula* taxa from *matK* inferred by RAxML. See Fig 3 for figure legends.
(DOCX)

S2 Fig Phylogenetic tree of *Pinguicula* taxa from *rpl32-trnL* inferred by RAxML. See Fig 3 for figure legends.
(DOCX)

S3 Fig Phylogenetic network of *Pinguicula* taxa from *matK* inferred by Neighbor-Net analysis. See Fig 6 for figure legends.
(DOCX)

S4 Fig Phylogenetic network of *Pinguicula* taxa from *rpl32-trnL* inferred by Neighbor-Net analysis. See Fig 6 for figure legends.
(DOCX)
