# Correction: Biogeographical patterns and speciation of the genus *Pinguicula* (Lentibulariaceae) inferred by phylogenetic analyses

**DOI:** 10.1371/journal.pone.0261600

**Published:** 2021-12-14

**Authors:** Hiro Shimai, Hiroaki Setoguchi, David L. Roberts, Miao Sun

Fig 5 is incorrect. The authors have provided a corrected version here.

**Fig 5:**
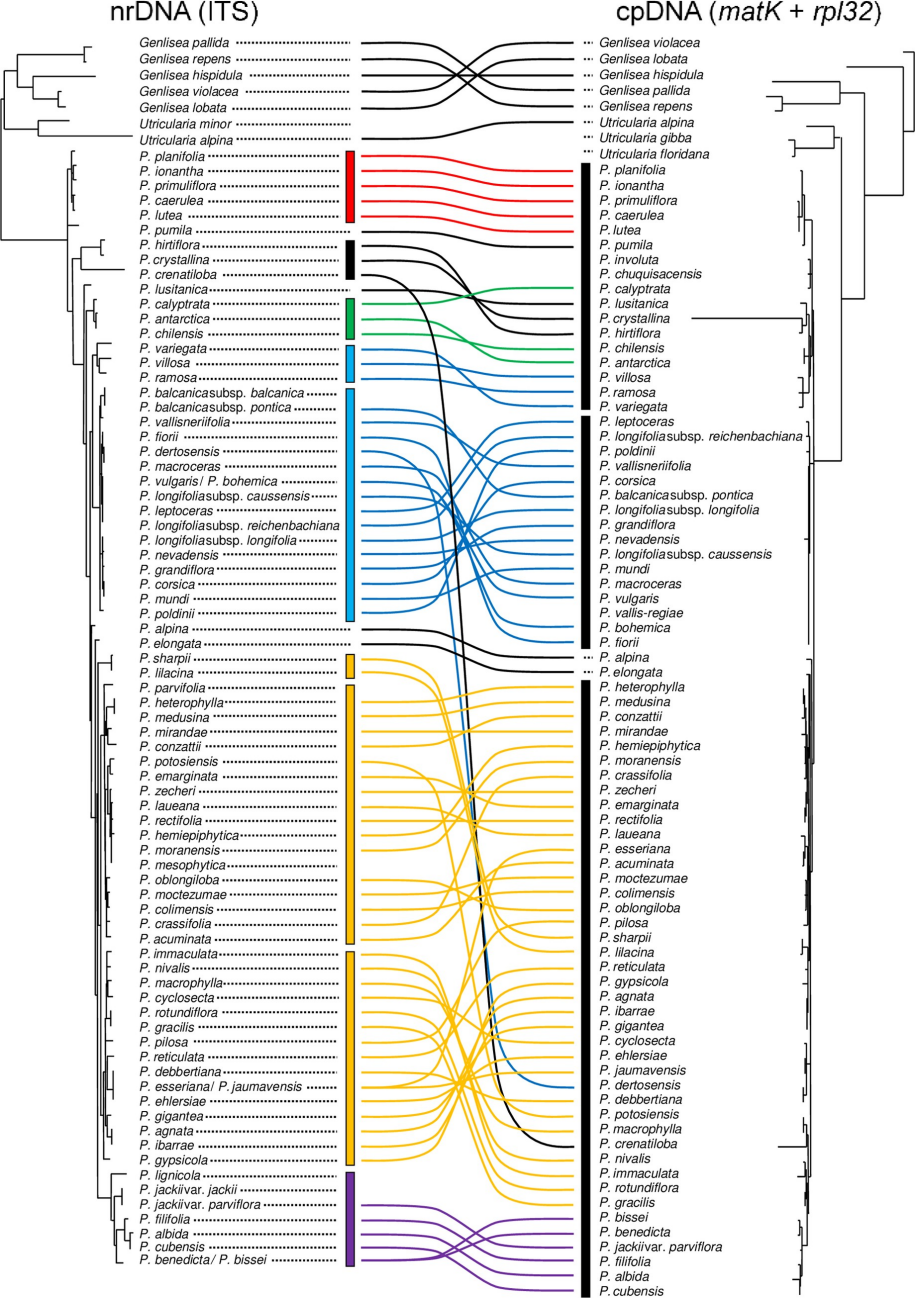
Phylogenetic comparison of nrDNA (ITS) and concatenated cpDNA. The figure shows topological incongruence between the ITS and combined cpDNA (*matK* + *rpl32-trnL*) trees. Vertical bars and connected lines are coloured based on major clades in the ITS tree; red for Clade I (the southeastern USA), green for Clade III (South America), blue for Clades IV and V (the temperate Northern Hemisphere), gold for Clades VI, VII, and VIII (Mexico and Central America), purple for Clade IX (Cuba), and black for others and the outgroup.
