# Supplementary material for: Biogeographical patterns and speciation of the genus Pinguicula (Lentibulariaceae) inferred by phylogenetic analyses
Source: PLoS One. 2021 Jun 7;16(6):e0252581. doi: 10.1371/journal.pone.0252581 (PMC8184156; doi:10.1371/journal.pone.0252581)
Supplement: S1 Appendix — (DOCX) [file pone.0252581.s001.docx]

**S1 Appendix. List of synonymous manes.**

Scientific names appearing in the article correspond with those in the International Nucleotide Sequence Database, but taxonomists may treat that some taxa are synonymous with others. Those representatives are listed here for reference purposes only.

*P. balcanica* subsp. *pontica* Casper = *P. balcanica* Casper

*P. bissei* Casper = *P. benedicta* Barnhart

*P. bohemica* Krajina = *P. vulgaris* L.

*P. chuquisacensis* S.Beck, A.Fleischm. & Borsch = *P. jarmilae* Halda & Malina

*P. ehlersiae* Speta & F.Fuchs = *P. esseriana* var. *ehlersiae* (Speta & F.Fuchs) Zamudio

*P. hirtiflora* Ten. = *P. crystallina* subsp. *hirtiflora* (Ten.) A.Strid

*P. jackii* var. *parviflora* Ernst = *P. lithophytica* Panfet & P.Temple

*P. jaumavensis* Debbert = *P. esseriana* B.Kirchn.

*P. longifolia* subsp. *caussensis* Casper = *P. caussensis* (Casper) Roccia

*P. longifolia* subsp. *reichenbachiana* (Schindler) Casper = *P. reichenbachiana* Schindler

*P. potosiensis* Speta & F.Fuchs = *P. moranensis* Kunth

*P. rectifolia* Speta & F.Fuchs = *P. moranensis* Kunth

*P. reticulata* Schlauer = *P. kondoi* Casper

*P. sharpii* Casper & K.Kondo = *P. lilacina* Schltdl. & Cham.

*P. variegata* Turcz. = *P. spathulata* Ledeb.

*P. zecheri* = Speta & F.Fuchs = *P. moranensis* Kunth
