# Supplementary material for: Biogeographical patterns and speciation of the genus Pinguicula (Lentibulariaceae) inferred by phylogenetic analyses
Source: PLoS One. 2021 Jun 7;16(6):e0252581. doi: 10.1371/journal.pone.0252581 (PMC8184156; doi:10.1371/journal.pone.0252581)
Supplement: S1 Fig — See Fig 3 for figure legends. (DOCX) [file pone.0252581.s002.docx]

**I**

**II**

**III**

**OG**

0.02
