# Supplementary material for: Biogeographical patterns and speciation of the genus Pinguicula (Lentibulariaceae) inferred by phylogenetic analyses
Source: PLoS One. 2021 Jun 7;16(6):e0252581. doi: 10.1371/journal.pone.0252581 (PMC8184156; doi:10.1371/journal.pone.0252581)
Supplement: Supplementary file 4 [file pone.0252581.s004.docx]

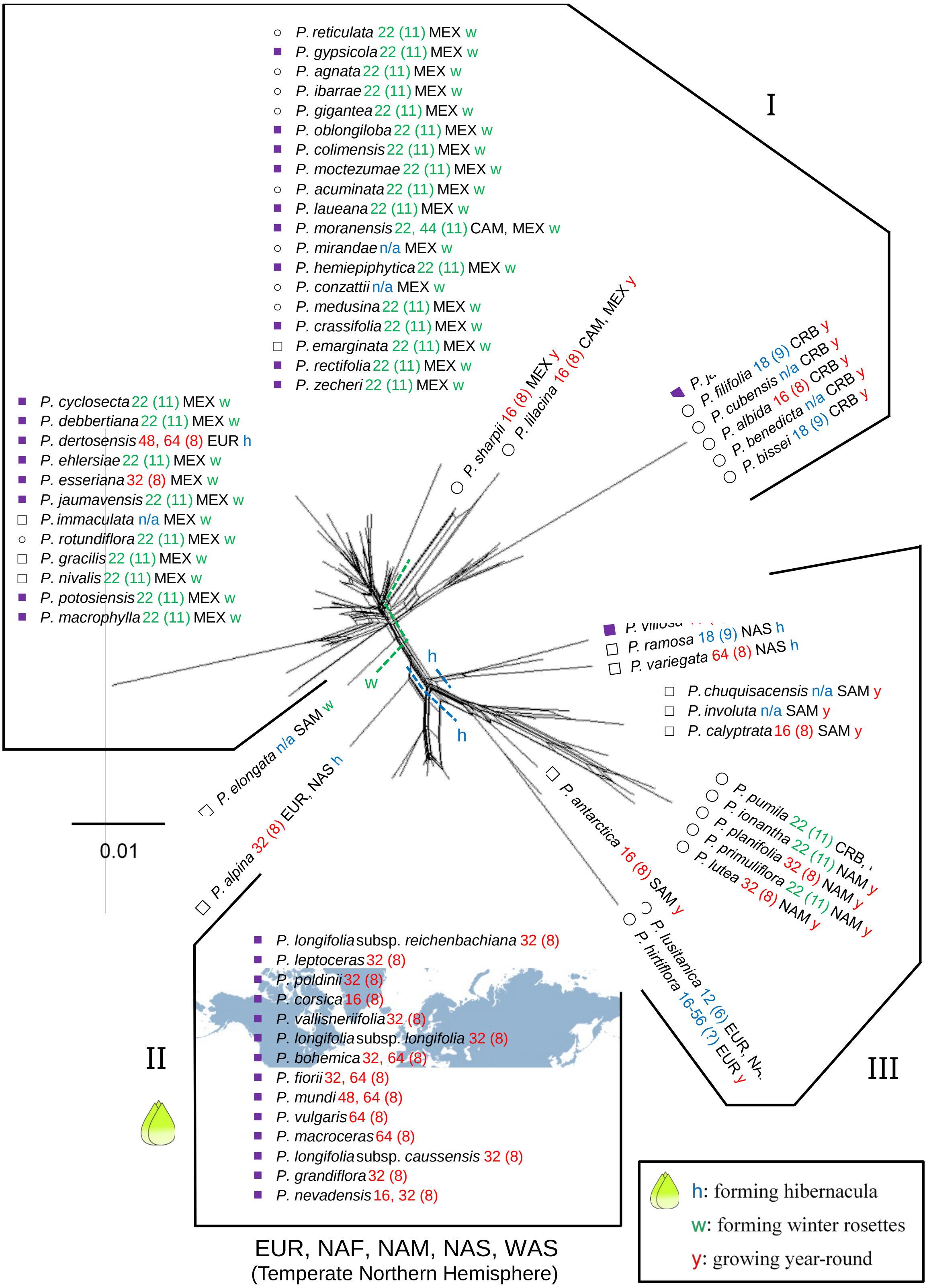

I
P. reticulata 22 (11) MEX w
P. gypsicola 22 (11) MEX w
P. agnata 22 (11) MEX w
P. ibarrae 22 (11) MEX w
P. gigantea 22 (11) MEX w
P. oblongiloba 22 (11) MEX w
P. colimensis 22 (11) MEX w
P. moctezumae 22 (11) MEX w
P. acuminata 22 (11) MEX w
P. laueana 22 (11) MEX w
P. moranensis 22, 44 (11) CAM, MEX w
P. mirandae n/a MEX w
P. hemiepiphytica 22 (11) MEX w
P. conzattii n/a MEX w
P. medusina 22 (11) MEX w
P. crassifolia 22 (11) MEX w
P. emarginata 22 (11) MEX w
P. rectifolia 22 (11) MEX w
P. zecheri 22 (11) MEX w
P. cyclosecta 22 (11) MEX w
P. debbertiana 22 (11) MEX w
P. dertosensis 48, 64 (8) EUR h
P. ehlersiae 22 (11) MEX w
P. esseriana 32 (8) MEX w
P. jaumavensis 22 (11) MEX w
P. immaculata n/a MEX w
P. rotundiflora 22 (11) MEX w
P. gracilis 22 (11) MEX w
P. nivalis 22 (11) MEX w
P. potosiensis 22 (11) MEX w
P. macrophylla 22 (11) MEX w
P. sharpii 16 (8) MEX y
P. lilacina 16 (8) CAM, MEX y
P. filifolia 18 (9) CRB y
P. cubensis n/a CRB y
P. albida 16 (8) CRB y
P. benedicta n/a CRB y
P. bissei 18 (9) CRB y
P. ramosa 18 (9) NAS h
P. variegata 64 (8) NAS h
P. chuquisacensis n/a SAM y
P. involuta n/a SAM y
P. calyptrata 16 (8) SAM y
P. elongata n/a SAM w
P. alpina 32 (8) EUR, NAS h
P. antarctica 16 (8) SAM y
P. pumila 22 (11) CRB,
P. ionantha 22 (11) NAM y
P. planifolia 32 (8) NAM y
P. primuliflora 22 (11) NAM y
P. lutea 32 (8) NAM y
P. lusitanica 12 (6) EUR,
P. hirtiflora 16-56 (?) EUR y
w
h
h
0.01
II
P. longifolia subsp. reichenbachiana 32 (8)
P. leptoceras 32 (8)
P. poldinii 32 (8)
P. corsica 16 (8)
P. vallisneriifolia 32 (8)
P. longifolia subsp. longifolia 32 (8)
P. bohemica 32, 64 (8)
P. fiorii 32, 64 (8)
P. mundi 48, 64 (8)
P. vulgaris 64 (8)
P. macroceras 64 (8)
P. longifolia subsp. caussensis 32 (8)
P. grandiflora 32 (8)
P. nevadensis 16, 32 (8)
EUR, NAF, NAM, NAS, WAS
(Temperate Northern Hemisphere)
III
h: forming hibernacula
w: forming winter rosettes
y: growing year-round
