# Supplementary material for: Biogeographical patterns and speciation of the genus Pinguicula (Lentibulariaceae) inferred by phylogenetic analyses
Source: PLoS One. 2021 Jun 7;16(6):e0252581. doi: 10.1371/journal.pone.0252581 (PMC8184156; doi:10.1371/journal.pone.0252581)
Supplement: Supplementary file 5 [file pone.0252581.s005.docx]

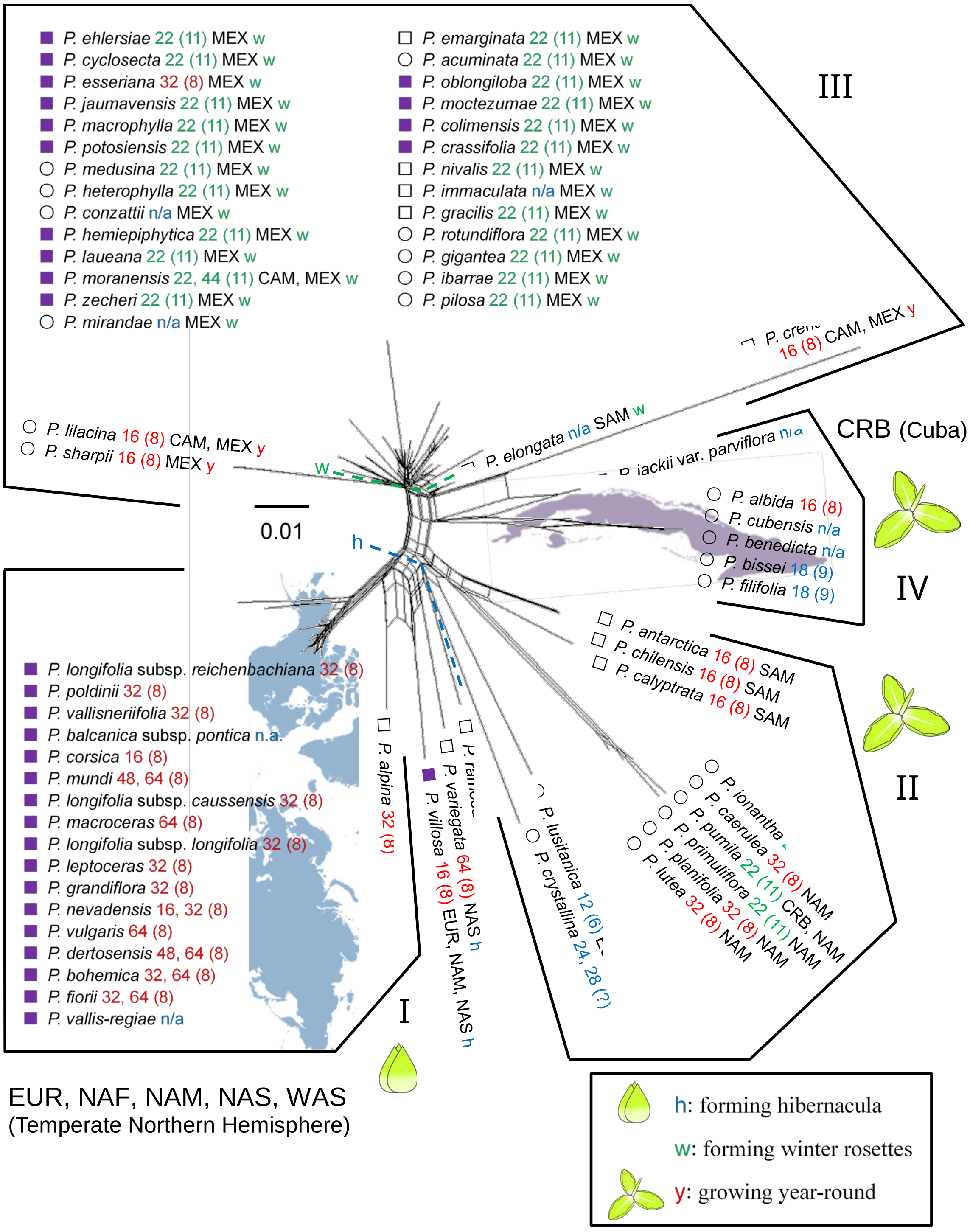

P. ehlersiae 22 (11) MEX w
P. cyclosecta 22 (11) MEX w
P. esseriana 32 (8) MEX w
P. jaumavensis 22 (11) MEX w
P. macrophylla 22 (11) MEX w
P. potosiensis 22 (11) MEX w
P. medusina 22 (11) MEX w
P. heterophylla 22 (11) MEX w
P. conzattii n/a MEX w
P. hemiepiphytica 22 (11) MEX w
P. laueana 22 (11) MEX w
P. moranensis 22, 44 (11) CAM, MEX w
P. zecheri 22 (11) MEX w
P. mirandae n/a MEX w
P. emarginata 22 (11) MEX w
P. acuminata 22 (11) MEX w
P. oblongiloba 22 (11) MEX w
P. moctezumae 22 (11) MEX w
P. colimensis 22 (11) MEX w
P. crassifolia 22 (11) MEX w
P. nivalis 22 (11) MEX w
P. immaculata n/a MEX w
P. gracilis 22 (11) MEX w
P. rotundiflora 22 (11) MEX w
P. gigantea 22 (11) MEX w
P. ibarrae 22 (11) MEX w
P. pilosa 22 (11) MEX w
III
16 (8) CAM, MEX y
P. lilacina 16 (8) CAM, MEX y
P. sharpii 16 (8) MEX y
P. elongata n/a SAM w
w
0.01
h
CRB (Cuba)
P. albida 16 (8)
P. cubensis n/a
P. benedicta n/a
P. bissei 18 (9)
P. filifolia 18 (9)
IV
P. antarctica 16 (8) SAM
P. chilensis 16 (8) SAM
P. calyptrata 16 (8) SAM
II
P. ionantha
P. caerulea 32 (8) NAM
P. pumila 22 (11) CRB, NAM
P. primuliflora 22 (11) NAM
P. planifolia 32 (8) NAM
P. lutea 32 (8) NAM
P. lusitanica 12 (6)
P. crystallina 24, 28 (?)
P. alpina 32 (8)
P. variegata 64 (8) NAS h
P. villosa 16 (8) EUR, NAM, NAS h
P. longifolia subsp. reichenbachiana 32 (8)
P. poldinii 32 (8)
P. vallisneriifolia 32 (8)
P. balcanica subsp. pontica n.a.
P. corsica 16 (8)
P. mundi 48, 64 (8)
P. longifolia subsp. caussensis 32 (8)
P. macroceras 64 (8)
P. longifolia subsp. longifolia 32 (8)
P. leptoceras 32 (8)
P. grandiflora 32 (8)
P. nevadensis 16, 32 (8)
P. vulgaris 64 (8)
P. dertosensis 48, 64 (8)
P. bohemica 32, 64 (8)
P. fiorii 32, 64 (8)
P. vallis-regiae n/a
I
EUR, NAF, NAM, NAS, WAS
(Temperate Northern Hemisphere)
h: forming hibernacula
w: forming winter rosettes
y: growing year-round
